# HemaScope: A Tool for Analyzing Single-cell and Spatial Transcriptomics Data of Hematopoietic Cells

**DOI:** 10.1093/gpbjnl/qzaf002

**Published:** 2025-01-25

**Authors:** Zhenyi Wang, Yuxin Miao, Hongjun Li, Wenyan Cheng, Minglei Shi, Gang Lv, Yating Zhu, Junyi Zhang, Tingting Tan, Jin Gu, Michael Q Zhang, Jianfeng Li, Hai Fang, Zhu Chen, Saijuan Chen

**Affiliations:** Shanghai Institute of Hematology, State Key Laboratory of Medical Genomics, National Research Center for Translational Medicine at Shanghai, Research Unit of Hematologic Malignancies Genomics and Translational Research of Chinese Academy of Medical Sciences, Ruijin Hospital, Shanghai Jiao Tong University School of Medicine, Shanghai 200025, China; MOE Key Laboratory of Bioinformatics, Beijing National Research Center for Information Science and Technology, Bioinformatics Division, Department of Automation, Tsinghua University, Beijing 100084, China; MOE Key Laboratory of Bioinformatics, Beijing National Research Center for Information Science and Technology, Bioinformatics Division, Department of Automation, Tsinghua University, Beijing 100084, China; Shanghai Institute of Hematology, State Key Laboratory of Medical Genomics, National Research Center for Translational Medicine at Shanghai, Research Unit of Hematologic Malignancies Genomics and Translational Research of Chinese Academy of Medical Sciences, Ruijin Hospital, Shanghai Jiao Tong University School of Medicine, Shanghai 200025, China; Institute of Medical Innovation and Research, Peking University Third Hospital, Beijing 100191, China; Shanghai Institute of Hematology, State Key Laboratory of Medical Genomics, National Research Center for Translational Medicine at Shanghai, Research Unit of Hematologic Malignancies Genomics and Translational Research of Chinese Academy of Medical Sciences, Ruijin Hospital, Shanghai Jiao Tong University School of Medicine, Shanghai 200025, China; School of Life Science and Technology, ShanghaiTech University, Shanghai 201210, China; Shanghai Institute of Hematology, State Key Laboratory of Medical Genomics, National Research Center for Translational Medicine at Shanghai, Research Unit of Hematologic Malignancies Genomics and Translational Research of Chinese Academy of Medical Sciences, Ruijin Hospital, Shanghai Jiao Tong University School of Medicine, Shanghai 200025, China; Shanghai Institute of Hematology, State Key Laboratory of Medical Genomics, National Research Center for Translational Medicine at Shanghai, Research Unit of Hematologic Malignancies Genomics and Translational Research of Chinese Academy of Medical Sciences, Ruijin Hospital, Shanghai Jiao Tong University School of Medicine, Shanghai 200025, China; MOE Key Laboratory of Bioinformatics, Beijing National Research Center for Information Science and Technology, Bioinformatics Division, Department of Automation, Tsinghua University, Beijing 100084, China; Department of Biological Sciences, Center for Systems Biology, The University of Texas at Dallas, Richardson, TX 75080-3021, USA; Shanghai Institute of Hematology, State Key Laboratory of Medical Genomics, National Research Center for Translational Medicine at Shanghai, Research Unit of Hematologic Malignancies Genomics and Translational Research of Chinese Academy of Medical Sciences, Ruijin Hospital, Shanghai Jiao Tong University School of Medicine, Shanghai 200025, China; Shanghai Institute of Hematology, State Key Laboratory of Medical Genomics, National Research Center for Translational Medicine at Shanghai, Research Unit of Hematologic Malignancies Genomics and Translational Research of Chinese Academy of Medical Sciences, Ruijin Hospital, Shanghai Jiao Tong University School of Medicine, Shanghai 200025, China; Shanghai Institute of Hematology, State Key Laboratory of Medical Genomics, National Research Center for Translational Medicine at Shanghai, Research Unit of Hematologic Malignancies Genomics and Translational Research of Chinese Academy of Medical Sciences, Ruijin Hospital, Shanghai Jiao Tong University School of Medicine, Shanghai 200025, China; Shanghai Institute of Hematology, State Key Laboratory of Medical Genomics, National Research Center for Translational Medicine at Shanghai, Research Unit of Hematologic Malignancies Genomics and Translational Research of Chinese Academy of Medical Sciences, Ruijin Hospital, Shanghai Jiao Tong University School of Medicine, Shanghai 200025, China

**Keywords:** scRNA-seq, Spatial transcriptomics, R package, Web server, Shiny

## Abstract

Single-cell RNA sequencing (scRNA-seq) and spatial transcriptomics (ST) techniques hold great value in evaluating the heterogeneity and spatial characteristics of hematopoietic cells within tissues. These two techniques are highly complementary, with scRNA-seq offering single-cell resolution and ST retaining spatial information. However, there is an urgent demand for well-organized and user-friendly toolkits capable of handling single-cell and spatial information. Here, we present HemaScope, a specialized bioinformatics toolkit featuring modular designs to analyze scRNA-seq and ST data generated from hematopoietic cells. It enables users to perform quality control, basic analysis, cell atlas construction, cellular heterogeneity exploration, and dynamical examination on scRNA-seq data. Also, it can perform spatial analysis and microenvironment analysis on ST data. Meanwhile, HemaScope takes into consideration hematopoietic cell-specific features, including lineage affiliation evaluation, cell cycle prediction, and marker gene collection. To enhance the user experience, we have deployed the toolkit in user-friendly forms: *HemaScopeR* (an R package), *HemaScopeCloud* (a web server), *HemaScopeDocker* (a Docker image), and *HemaScopeShiny* (a graphical interface). In case studies, we employed it to construct a cell atlas of human bone marrow, analyze age-related changes, and identify acute myeloid leukemia cells in mice. Moreover, we characterized the microenvironments in angioimmunoblastic T cell lymphoma and primary central nervous system lymphoma, elucidating tumor boundaries. HemaScope is freely available at https://zhenyiwangthu.github.io/HemaScope_Tutorial/.

## Graphical abstract

**Graphical abstract:**
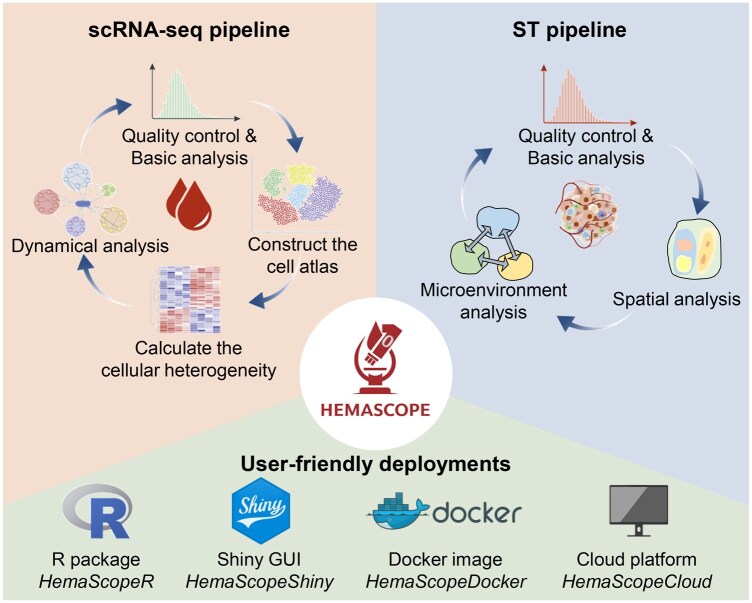

## Introduction

Single-cell RNA sequencing (scRNA-seq) [1] and spatial transcriptomics (ST) [2] techniques have emerged as pivotal methods for studying cellular heterogeneity and dynamic processes [3], as well as tissue spatial architecture. scRNA-seq offers high-throughput measurement of messenger RNA (mRNA) abundance in individual cells, allowing for gene expression profiling across numerous cells within a sample. Although ST can detect the transcriptome of specific spots with a defined resolution (*e.g.*, 10X Visium, 55 µm in diameter, and 100 µm center-to-center distance between spots), it sacrifices single-cell resolution. However, it can record spatial locations for each spot, making scRNA-seq data and ST data complementary.

With the advancements in computational methods, there are over 1400 algorithms currently available to analyze scRNA-seq and ST data [4,5]. However, there are many pitfalls and recommendations that should be considered in data analysis practices [6]. These present challenges in choosing appropriate algorithms and designing effective data analysis pipelines. In previous studies, various research scope-specific scRNA-seq and ST analysis toolkits have been reported. For example, Guo et al. introduced scCancer [7], a specialized tool designed for processing scRNA-seq data in cancer research. Prieto et al. developed SingleCAnalyzer [8], a user-friendly cloud-based platform for comprehensive scRNA-seq analysis from FAST-Quality (FASTQ) files. SINCERA [9], developed by Guo et al., focused on processing scRNA-seq data from sorted cells or entire organs. Dries et al. introduced Giotto [10], an open-source toolbox for spatial data analysis, while Palla et al. presented Squidpy [11], a Python framework that merges omics and image analysis for scalable interpretation of spatial molecular data. Despite these popular toolkits making data analysis in several research scopes more convenient and efficient, there remains a pressing need for toolkits specific to hematopoietic cells that also have user-friendly features adaptable to varying computational skill levels. We compared 20 milestone toolkits in detail with the one presented in this work in Table S1.

In this work, we developed a modular computational toolkit tailored for in-depth analysis of scRNA-seq and ST data from hematopoietic cells. HemaScope embodies the principles of portability, reproducibility, scalability, and user-friendliness. It encompasses quality control for scRNA-seq and ST data, and performs both unsupervised and supervised downstream analyses. For scRNA-seq data, it offers a suite of functionalities, including clustering, cell type identification, copy number variation (CNV) estimation, detection of differentially expressed genes (DEGs), cell cycle analysis, heterogeneity quantification, lineage differentiation potential assessment, cell trajectory prediction, transcription factor (TF) analysis, gene set variation analysis (GSVA), and cell–cell interaction analysis. For ST data, it includes detection of spatially variable features, deconvolution, coexistence analysis, and spatial interaction analysis. To enhance the user experience, we developed an open-source R package named “*HemaScopeR*” with an intuitive graphical interface named “*HemaScopeShiny*”, a web server named “*HemaScopeCloud*”, a Docker image named “*HemaScopeDocker*”, and comprehensive step-by-step tutorials.

Aside from the technical aspects above, HemaScope also takes special consideration of hematopoietic cell-specific features. First, HemaScope introduces comprehensive quality control metrics. Second, its downstream analysis targets critical issues in hematopoietic cell research, including cellular heterogeneity, dynamics, hematopoietic hierarchy, and niches. Third, it introduces a lineage score to measure the affiliation levels of cells with various lineages within the hematopoietic hierarchy. Additionally, it introduces a strategy to estimate the cell cycle, and combines hematopoietic marker gene expression, data-driven prediction, and artificial intelligence-based methods to annotate hematopoietic cell types. Furthermore, it integrates the latest visual representation of the cellular hematopoietic hierarchy presented by Dai et al. (https://github.com/NRCTM-bioinfo/HematoMap), allowing researchers to visually observe hematopoiesis cellular composition and the cell blockage of acute leukemia in data through intuitive tree diagrams. By leveraging HemaScope, we analyzed five publicly available datasets, systematically demonstrating its efficacy across these analysis tasks.

## Method

### HemaScope overview

**Figure 1:**
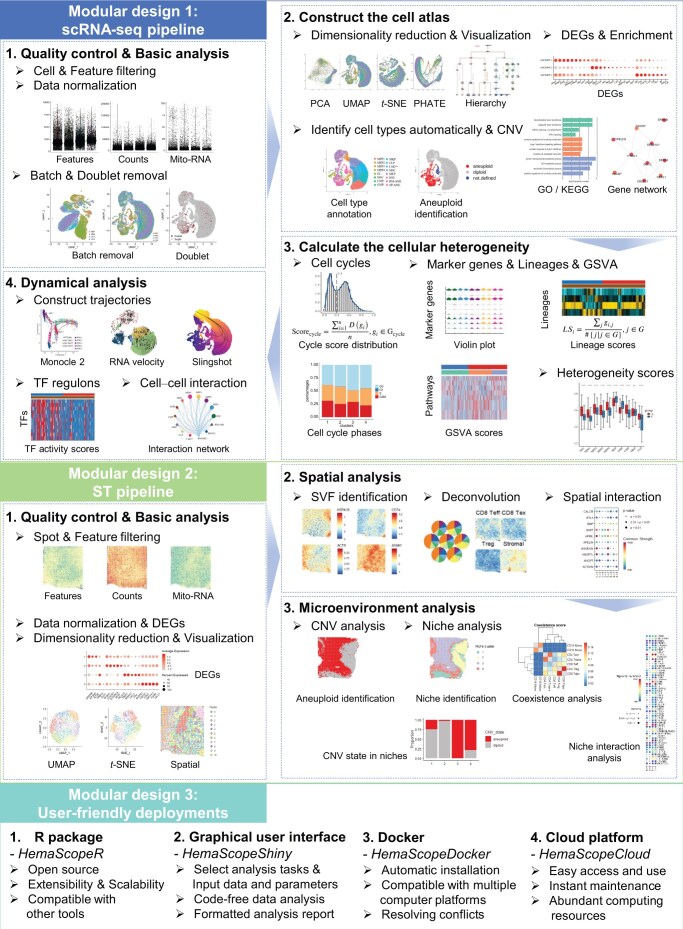
Overview of the HemaScope toolkit, featuring its three modular designs scRNA-seq, single-cell RNA sequencing; PCA, principal component analysis; UMAP, Uniform Manifold Approximation and Projection; *t*-SNE, *t*-distributed Stochastic Neighbor Embedding; PHATE, Potential of Heat-diffusion for Affinity-based Trajectory Embedding; DEG, differentially expressed gene; CNV, copy number variation; GO, Gene Ontology; KEGG, Kyoto Encyclopedia of Genes and Genomes; GSVA, gene set variation analysis; TF, transcription factor; ST, spatial transcriptomics; SVF, spatially variable feature.

As illustrated in **Figure 1**, the HemaScope toolkit consists of three modules. The first module covers the analytical workflow for scRNA-seq data, comprising four major components: data quality control and basic analysis, construction of a cell atlas, calculation of cellular heterogeneity, and examination of dynamic characteristics within cell populations. The second module is dedicated to the analytical pipeline for ST data, encompassing data quality control and basic analysis, spatial analysis, and microenvironment analysis. The third module includes four user-friendly features. We developed an R package with a graphical user interface that enables users to execute analyses either by coding command lines or by clicking buttons. Additionally, we distributed a Docker image to streamline the runtime environment setup and software dependencies. To accommodate the diverse needs of researchers, we also developed an online cloud platform. All bioinformatics tools integrated into HemaScope are listed in Table S2.

### Modular design 1: scRNA-seq pipeline

This pipeline accepts input from either the processed output files generated by 10X Genomics Cell Ranger, a Seurat [12] object, or a gene expression matrix in which rows denote genes and columns denote cells. It consists of four primary components.

#### The first part includes quality control and basic analysis

Initially, cells undergo filtering based on specific criteria: (1) cells expressing fewer than the specified “min.feature” number of genes, and (2) cells exceeding the “percent.mt.limit” for mitochondrial count percentage. Only genes detected in at least “min.cells” cells are retained. Subsequently, gene expression values are normalized, variable features are identified for principal component analysis (PCA), and DoubletFinder [13] is employed to identify doublets. Additionally, batch effects are mitigated using the FindIntegrationAnchors strategy from Seurat. We provided pitfalls and recommendations for setting cutoffs in File S1.

#### The second part is dedicated to constructing the cell atlas

Cells are visualized in 2-dimensional (2D) space using dimensionality reduction methods, including PCA [14], Uniform Manifold Approximation and Projection (UMAP) [15], *t*-distributed Stochastic Neighbor Embedding (*t*-SNE) [16], Potential of Heat-diffusion for Affinity-based Trajectory Embedding (PHATE) [17], and a tree-like structure by HematoMap (details are provided in File S1 and Figure S1). Subsequently, the Louvain clustering algorithm [18] is employed for unsupervised cell cluster identification. Next, seven methods are integrated to improve the accuracy of cell type annotation (details are provided in File S1). For the convenience of readers, we referred to the literature [19–23] and online data resources [24], and provided well-organized marker gene lists for human and mouse hematopoietic cells in Tables S3 and S4. The Wilcoxon rank-sum test is then utilized to identify highly expressed gene sets within each cell type, followed by Gene Ontology (GO) and Kyoto Encyclopedia of Genes and Genomes (KEGG) pathway enrichment analysis using clusterProfiler [25], as well as gene network analysis via OpenXGR [26] to explore the features of each cell type from different perspectives.

#### The third part focuses on calculating cellular heterogeneity

To quantify the heterogeneity of hematopoietic cells, we designed specific parameters and collected gene sets. An emerging theme in hematopoietic stem cell (HSC) and leukemia research is the equilibrium between cell proliferation and quiescence within the HSC pool through the cell cycle [27,28]. In HemaScope, we referred to the work of Dong et al. [20] to further introduce a parameter named cell cycle score (*Score_cycle_*), combined with a data-driven machine learning method, scran [29], to classify single cells to G0, G1, S, and G2M phases (details are in File S1 and Table S5). We also designed a parameter called lineage score (*LS_i_*) which can quantify the affiliation levels of individual cells to various lineages within the hematopoietic hierarchy based on the gene expression profiles (details are in File S1; Tables S6 and S7). *LS_i_* not only indicates the potential of normal hematopoietic stem/progenitor cells to differentiate into different lineages, but also detects cell blockage in leukemia. Then, cellular heterogeneity within cell groups is assessed by using the Spearman correlation coefficient, measuring differences among single cells. The Wilcoxon rank-sum test evaluates the statistical significance of average similarity differences between two sample groups. Next, expert annotated gene sets from the Molecular Signatures Database (MSigDB) and GSVA [30] are employed to explore the enriched biological processes in each cell.

#### The fourth part involves dynamical analysis

HemaScope is capable of analyzing cellular dynamics from three perspectives: cell trajectory, TF regulation, and cell–cell communication. Monocle 2 [31] is used to analyze cell differentiation trajectories, employing the minimum spanning tree approach. Slingshot [32] is utilized to investigate differentiation trajectories based on PCA. Additionally, scVelo [33] is employed to analyze cell differentiation trajectories based on RNA velocity. The results obtained from these three analytical methods, each grounded in distinct model assumptions, provide complementary insights. We compared these three methods in terms of algorithmic principles in File S1. Following the aforementioned analyses, SCENIC [34] is used to compute activated TF regulons in each cell, followed by CellChat [35], which assesses the cross-talk among different cell populations.

### Modular design 2: ST pipeline

The ST pipeline in HemaScope is capable of processing spatial transcriptomes with various resolutions generated by diverse spatial molecular technologies, including 10X Visium, multiplexed error-robust fluorescence *in situ* hybridization (MERFISH), and spatial enhanced resolution omics-sequencing (Stereo-seq). This pipeline comprises three primary parts.

#### The first part comprises quality control and basic analysis

Upon data loading, basic quality control measures are implemented. Cells with low unique molecular identifier (UMI) counts or gene numbers, as well as genes with minimal occurrence, are filtered out. Subsequently, Seurat [36] is employed to normalize, scale (or apply SCTransform), and perform PCA on the refined dataset, resulting in a low-dimensional representation defined by the first 10 principal components (PCs) by default. These PCs are then used for shared-nearest-neighbor (SNN)-based clustering, as well as for generating UMAP and *t*-SNE visualizations. DEGs across all clusters are identified based on fold changes and adjusted *P* values, determined via the Wilcoxon rank-sum test with Bonferroni correction.

#### The second part engages in spatial analysis

HemaScope incorporates the detection of spatially variable features (SVFs), spatial interaction analysis, and deconvolution. SVFs are identified using the “FindSpatiallyVariableFeatures” function in Seurat, which employs Global Moran’s I. Communication analysis by optimal transport (COMMOT) [37] is then utilized to analyze intercellular spatial communication. By default, secreted signaling ligand–receptor pairs from the CellChatDB [35] database are utilized to infer spatial interaction strength. These significantly expressed ligand–receptor pairs within and between clusters are visualized using dot plots. In the deconvolution step, HemaScope infers the abundance distribution of various cell types from mixed signals in 10X Visium data using cell2location. The resulting cell type distribution across the slide serves as the basis for microenvironment analysis. Additionally, reference single-cell datasets are utilized to calculate the top 50 DEGs for each cell type as marker genes. These marker genes are used to score each cell type, complementing the results from cell2location (details are provided in File S1).

#### The third part focuses on microenvironment analysis

It starts with CNV analysis to pinpoint tumor areas in tissues, employing copy number karyotyping of aneuploid tumors (CopyKAT) [38] to distinguish tumor regions from normal ones within the microenvironment. Following CNV analysis, the coexistence of various cell types in the tissue is calculated based on deconvolution results. To assess the global coexistence of cell types, correlation or Wasserstein distance is used to calculate their distributional distance. The Pearson correlation [39] measures the correlation-based coexistence score, while the Wasserstein distance defines the distribution-based coexistence score for cell types (details are provided in File S1). Next, diverse niches are identified using *K*-means clustering based on the absolute cellular abundances from deconvolution results. The cellular abundance of these niches and the proportion of tumor regions within each niche aid researchers in evaluating niche characteristics. Furthermore, COMMOT infers and visualizes interactions within and between niches.

### Modular design 3: user-friendly deployments

**Figure 2:**
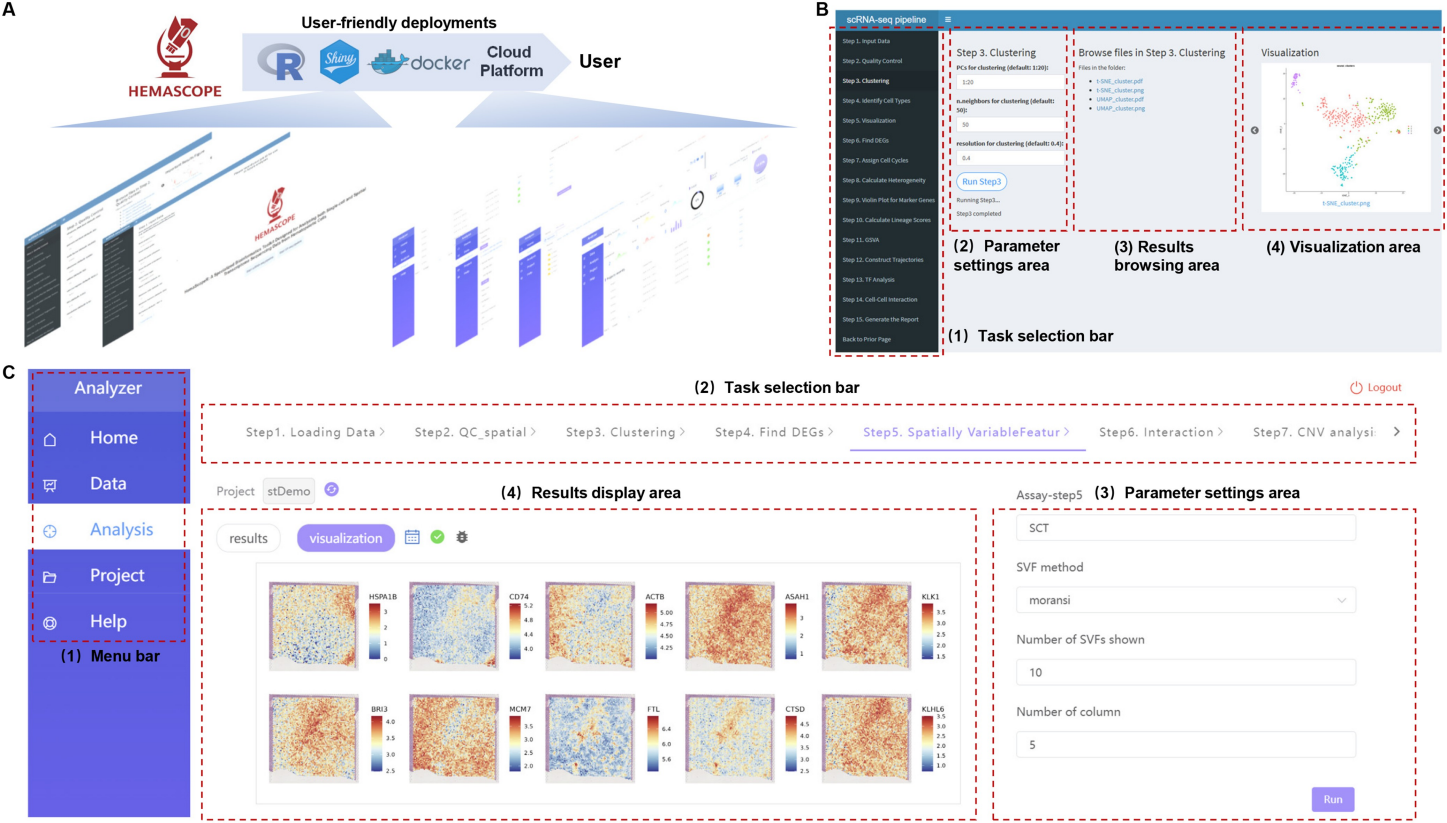
User-friendly deployments of HemaScope **A**. HemaScope includes *HemaScopeR* (an R package), *HemaScopeShiny* (a Shiny GUI), *HemaScopeDocker* (a Docker image), and *HemaScopeCloud* (a cloud platform). **B**. A screenshot of *HemaScopeShiny* demonstrates features such as the task selection bar, the parameter settings area, the results browsing area, and the visualization area. **C**. A screenshot of *HemaScopeCloud* illustrates the menu bar, the task selection bar, the parameter settings area, and the results display area. GUI, Graphical User Interface.

As illustrated in **Figure 2**A, we developed four user-friendly features to enhance the user experience. First, *HemaScopeR* is a well-organized R package. This open-source package ensures high scalability and compatibility with diverse R packages. Second, *HemaScopeShiny* is a Graphical User Interface (GUI) based on Shiny [40]. As illustrated in Figure 2B, researchers can opt for the GUI mode when using *HemaScopeR*, enabling code-free data analysis. Third, for easier installation and deployment on local computers, we developed a Docker image, *HemaScopeDocker*, to automate software downloads, configure necessary toolkits, and ensure compatibility across various computing platforms. Fourth, we developed a cloud platform, *HemaScopeCloud* (Figure 2C), to facilitate researchers in conducting online data analyses. In Table S1, we provided a detailed comparison of our toolkit with 20 other milestones.

### Public datasets used for demo

To demonstrate the practical application capabilities of HemaScope in both a healthy population and various hematological diseases, we utilized publicly available scRNA-seq and ST datasets as case studies in the following sections and File S1. These datasets include scRNA-seq data of bone marrow cells from 20 healthy adult human donors across a broad age range [41], 45 acute myeloid leukemia (AML) patients [42,43], and scRNA-seq data of bone marrow cells from mice with *Myc*-driven AML [44], as well as scRNA-seq and ST data from primary central nervous system lymphoma (PCNSL) tumor samples [45] and ST data from angioimmunoblastic T cell lymphoma (AITL) samples [46]. We provide detailed information about these datasets in Table S8.

## Results

### HemaScope generates a comprehensive single-cell atlas of human bone marrow across a broad age range

**Figure 3:**
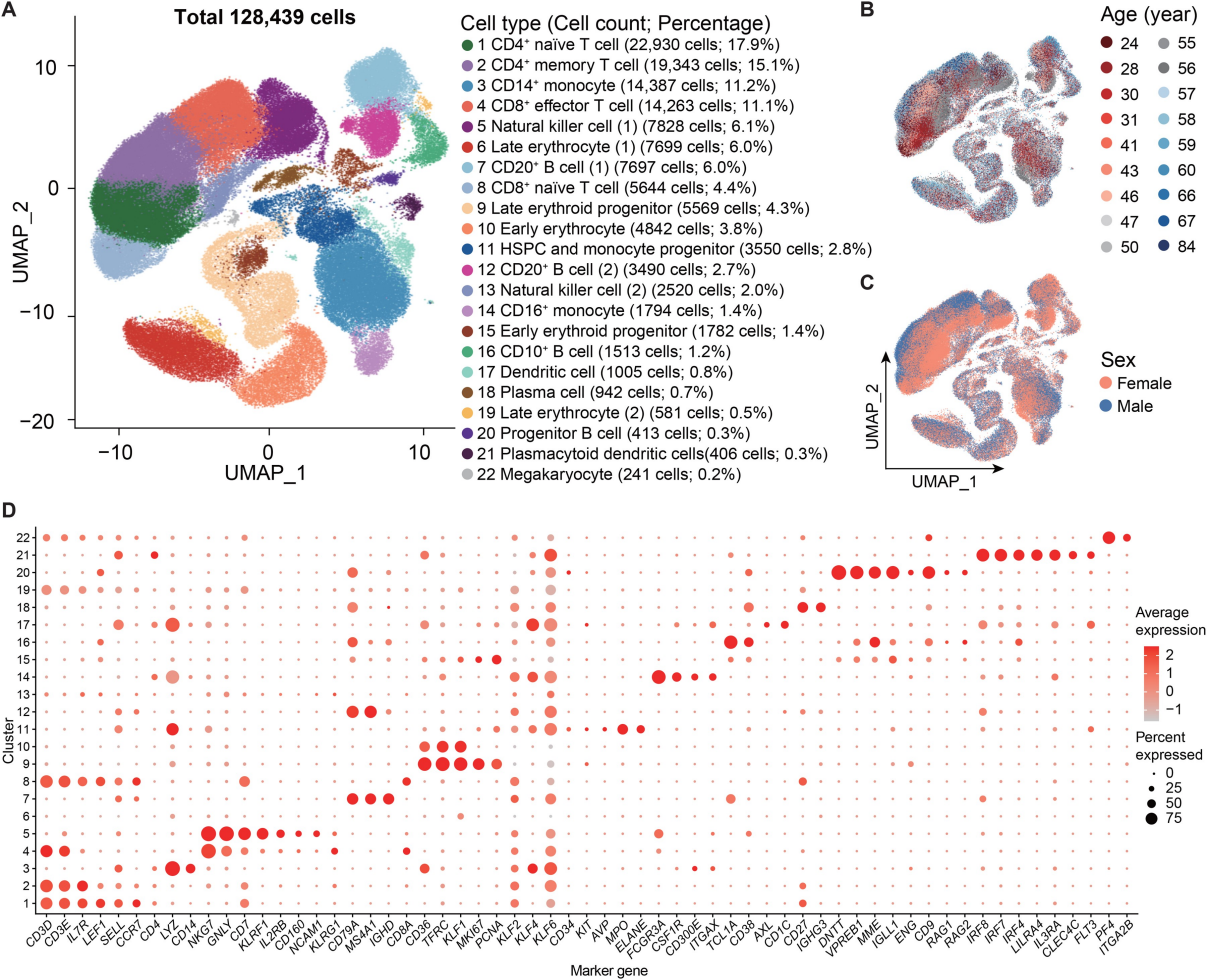
The single-cell atlas of human bone marrow across a broad age range **A**. Visualization of cell clusters in a two-dimensional space using UMAP, annotated by cell types, cell counts, and percentages (rounded to one decimal place). **B**. Age labels of 20 donors (ranging from 24 to 84 years) are represented by distinct colors of points. **C**. Sex labels of 20 donors, with red and blue points indicating males and females, respectively. **D**. Dot plot showcasing the marker genes of various cell types in bone marrow. Larger dots indicate a higher proportion of cells expressing the gene, while a deeper red hue indicates increased average expression within the specific cell type.

We analyzed scRNA-seq data obtained from bone marrow samples of 20 healthy donors aged between 24 and 84 years [41] using HemaScope. **Figure 3**A demonstrates the presence of 22 cell types, as well as cell counts and percentages, in the cell atlas generated by HemaScope. The statistical results are provided in Table S9. Figure S2A displays the distribution of gene numbers, read counts, and mitochondrial RNA proportion used in the quality control process, while the effective identification and removal of doublets are illustrated in Figure S2B using DoubletFinder within HemaScope. Notably, no significant batch effects were observed among the 20 donors’ data (Figure 3B and C, Figure S2C).

Identifying cell types in scRNA-seq data analysis is a challenging and open question. Inspired by the AdaBoost method [47] from the field of machine learning, we integrated seven methods to improve the accuracy of cell type prediction. These methods include profiling of hematopoietic cell-specific marker gene expression by various visualization strategies (Figure 3D, Figure S2D–F; marker genes are listed in Table S3), DEGs (Figure S2G; Table S10), GO enrichment, KEGG pathway enrichment, and gene network analysis (*e.g.*, Figure S2H displays the enriched gene network of DEGs in CD10^+^ B cells, *i.e.*, cluster 16 in Figure 3A), in addition to assessing ChatGPT [48] and label transfer (Figure S2I–O). We provided the technical details of the cell type annotation process in File S1. As depicted in Figure S2P, while there is considerable heterogeneity among donors, noticeable alterations in cell type proportions within the bone marrow were observed with increasing age. In particular, a significant increase in CD4^+^ naïve T cells and CD4^+^ memory T cells was evident [41], as shown in the stacked area plot. The computational details and statistical results on cell types, cell counts, and percentages are provided in File S1 and Table S9. Additionally, we employed HemaScope to dissect cellular heterogeneity and dynamics in human bone marrow cells, with further details provided in File S1 and Figure S3.

### HemaScope analyzes the microenvironment in PCNSL from a spatial perspective

**Figure 4:**
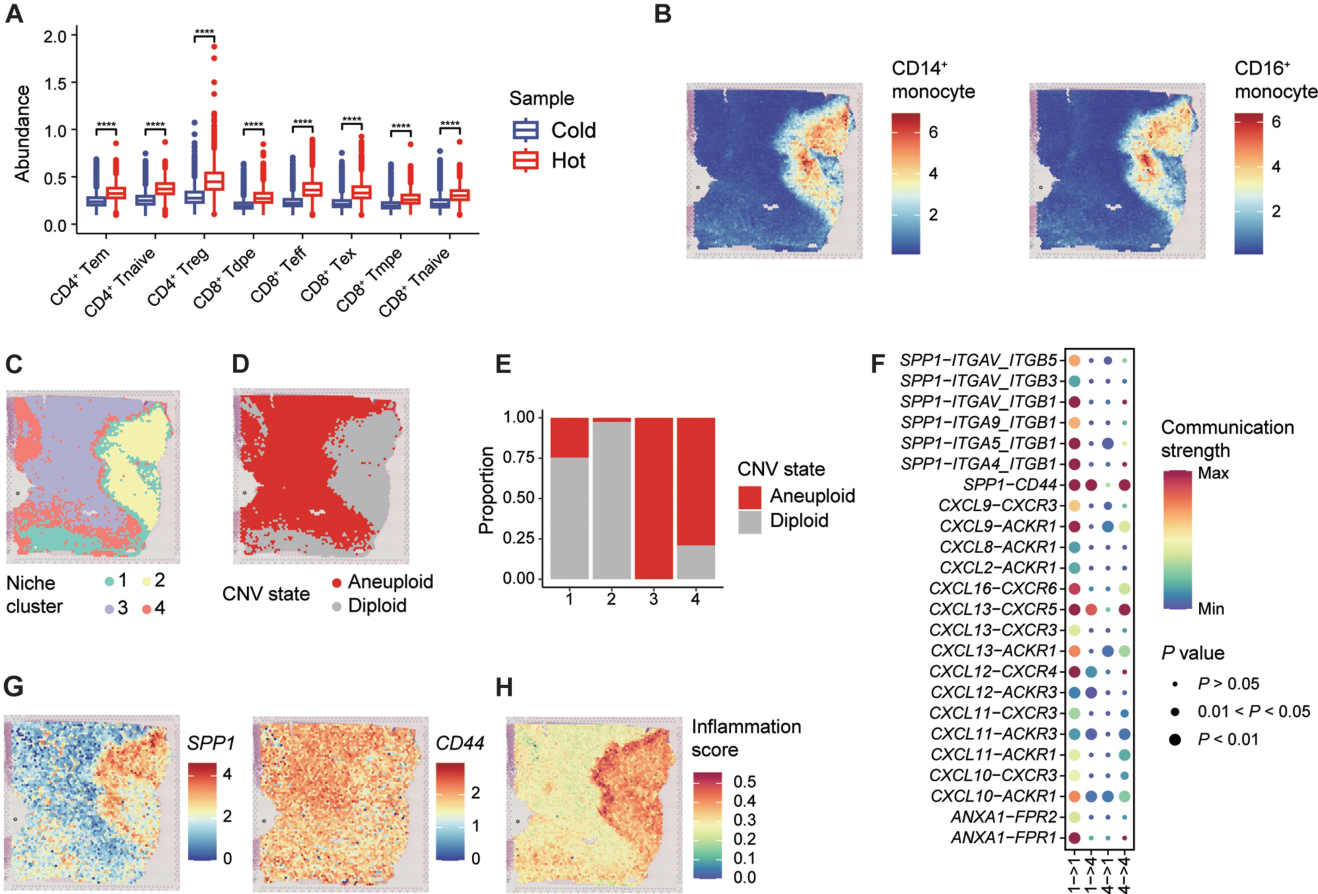
HemaScope analysis of the microenvironment in PCNSL **A**. Box plot depicting T cell abundance in “Hot” and “Cold” samples, with *P* values calculated using the Wilcoxon rank-sum test (****, *P* < 0.0001). **B**. Spatial representation of CD14^+^ and CD16^+^ monocyte abundance in the IME. **C**. Spatial representation of niche clusters. **D**. Spatial representation of CNV states inferred via CopyKAT. **E**. Bar chart presenting proportions of various CNV states within each niche cluster. **F**. Bubble heatmap indicating the strength and statistical significance of interactions within and between niche clusters 1 and 4. **G**. Spatial distribution of expression patterns of *SPP1* and *CD44*. **H**. Spatial representation of the inflammation scores. CD4^+^ Tem, CD4^+^ effector memory T cell; CD4^+^ Tnaive, CD4^+^ naïve T cell; CD4^+^ Treg, CD4^+^ regulatory T cell; CD8^+^ Tdpe, CD8^+^  *KLRG1*^+^  *IL7R*^+^ double-positive effector T cell; CD8^+^ Teff, CD8^+^ effector T cell; CD8^+^ Tex, CD8^+^ exhausted T cell; CD8^+^ Tmpe, CD8^+^ memory precursor effector T cell; CD8^+^ Tnaive, CD8^+^ naïve T cell; PCNSL, primary central nervous system lymphoma; IME, invasive margin-excluded; CopyKAT, copy number karyotyping of aneuploid tumors.

In this case, we used HemaScope to analyze the tumor microenvironment of four ST samples from PCNSL [45]. Analyzing the cellular composition and interactions in the microenvironment aids in understanding niche function [49]. Therefore, after basic quality control, deconvolution was conducted on the ST data using scRNA-seq data from HematoMap as a reference dataset. The “Hot” sample demonstrates a significantly higher infiltration level of T cells compared to the “Cold” sample (**Figure 4**A, Figure S4A and B), aligning with the inherent characteristics of each sample. Additionally, the non-tumor area of the invasive margin-excluded (IME) sample exhibits significant enrichment of CD14^+^ and CD16^+^ monocytes (Figure 4B), which is consistent with the macrophage definition in the original study [45].

Next, we categorized microenvironments by applying *K*-means clustering to the cellular abundance of these spatial data. The results highlighted the significance of the *CXCL12–CXCR4* pathway in “Hot” samples (Figure S4C and D), aligning with the findings of the original study [45]. In the IME sample, four microenvironment niches were identified (Figure 4C). Upon assessing the CNV results in the IME sample (Figure 4D), the analysis of spot CNV states across the four clusters indicated that clusters 1 and 2 were predominantly located in the non-tumor area, while clusters 3 and 4 were primarily found in the tumor area (Figure 4E). When examining the microenvironment cell composition within these clusters, clusters 1 and 2 exhibited an enrichment of monocytes and stromal cells, whereas clusters 3 and 4 displayed the opposite pattern (Figure S4E). Additionally, the upregulation of the *ANXA1–FPR1* signaling pathway in clusters 1 and 2 was consistent with the findings of the original study [45] (Figure S4F).

Furthermore, we performed an in-depth analysis of the interaction between cluster 1 and cluster 4, as they encompassed both tumor and non-tumor regions (Figure 4E). Notably, these microenvironments were situated at the boundary between tumor and non-tumor regions in the IME sample (Figure 4C and D). At this boundary, there was extensive activation of the *SPP1* signaling pathway, especially the *SPP1–CD44* signal (Figure 4F). A significant interaction between *SPP1* in cluster 1 and *CD44* in cluster 4 was observed, consistent with the high *SPP1* and *CD44* expression at the tumor boundary (Figure 4G, Figure S4G and H). Gene expression patterns indicated *SPP1* concentration in the non-tumor area, specifically within the region previously identified as containing macrophages. In various cancers, tumor-associated macrophage (TAM) expressing *SPP1* (TAM-*SPP1*) facilitated immune suppression and tumor progression by interacting with *CD44* receptors on tumor cells [50–53]. This suggested potential enrichment of TAM-*SPP1* at the PCNSL boundary, contributing to an immune-suppressive microenvironment. Meanwhile, the activation of the *CXCL13–CXCR5* signaling axis was observed within and between clusters 1 and 4 (Figure 4F, Figure S4I), indicating an inflammatory response [54] at the tumor boundary. Utilizing the inflammation features from CancerSEA [55], we scored the IME sample and revealed higher inflammation scores at the boundary (Figure 4H), corresponding to the activation of the *CXCL13–CXCR5* axis. Similar tumor boundary characteristics were also found in the invasive margin-immunosuppressed (IMS) sample (see File S1). In addition, we estimated spatial interactions between B cells and malignant T cells in an AITL sample using HemaScope (see File S1). In summary, we employed HemaScope to comprehensively delineate the characteristics of the tumor microenvironments in PCNSL and AITL.

## Discussion

HemaScope is a user-friendly and modular design toolkit tailored for analyzing single-cell and ST data of hematopoietic cells. It automates the analysis workflow by utilizing scRNA-seq or ST matrices and spot coordinates as inputs, generating publication-quality figures, well-formatted tables, and organized analysis reports. Our open-source R package, *HemaScopeR*, is highly extensible and compatible with other toolkits. To enhance the user experience, we developed a GUI (*HemaScopeShiny*) for code-free data analysis, a Docker image (*HemaScopeDocker*) for easy installation, and a web server (*HemaScopeCloud*) for online use.

The scRNA-seq pipeline consists of four primary components: quality control and basic analysis, construction of a cell atlas, evaluation of cellular heterogeneity, and dynamical analysis. First, we conducted quality control, dimensionality reduction, visualization, cell type identification, CNV detection, DEG analysis, GO/KEGG pathway enrichment, and gene network analysis to aid in building a comprehensive cell atlas. Next, we evaluated cellular heterogeneity and dynamics by predicting cell cycle phases (G0, G1, S, and G2M) and quantifying lineage affiliation. We then applied the GSVA algorithm to analyze cell enrichment in gene sets from the MSigDB and used Spearman correlation coefficients to assess intercellular heterogeneity. For the dynamical analysis, we employed Monocle 2, scVelo, and Slingshot to predict cellular trajectories. Additionally, we used SCENIC to investigate TF regulons and CellChat to explore cell–cell interactions. HemaScope was utilized to construct a cell atlas, track age-related changes, and investigate cellular heterogeneity and dynamics in human bone marrow, as well as to identify preleukemic and leukemic cells in mouse bone marrow. The effectiveness of this scRNA-seq pipeline is supported by literature validation.

Characterizing the tumor microenvironment from a spatial perspective is essential for understanding tumor progression within the ST pipeline. In addition to core analyses, HemaScope focuses on examining cellular composition, CNV status, and spatial interactions to provide a comprehensive view of the tumor microenvironment. Through the analysis of five spatial transcriptome samples from PCNSL and AITL, HemaScope not only validated the findings of the original study [45] but also offered deeper insights, particularly regarding the tumor boundary. The results suggested a significant accumulation of TAM-*SPP1* at the PCNSL tumor boundary, contributing to an immunosuppressive environment, along with evidence of a marked inflammatory response in this region.

In the future, there are several aspects where HemaScope can be further enhanced. First, single-cell multi-omics technologies [56] enable the simultaneous acquisition of transcriptomic, proteomic, and chromatin accessibility data at single-cell resolution, highlighting the need for pipelines that can integrate these diverse data types. Second, as the resolution of ST continues to improve [57], toolkits must be developed to scale and adapt to varying resolutions. Third, the rise of spatial multi-omics technologies [58] creates a demand for pipelines capable of handling spatial multi-omics data. Additionally, with the continuous development of new algorithms and the expanding knowledge of hematopoietic cells, these advancements should be incorporated into HemaScope to further optimize the toolkit, ensuring both user-friendliness and flexibility (File S1).

## Code availability

The open-source R package “*HemaScopeR*”, along with the GUI “*HemaScopeShiny*”, is available online at GitHub (https://github.com/ZhenyiWangTHU/HemaScopeR/). The code has also been submitted to BioCode at the National Genomics Data Center (NGDC), China National Center for Bioinformation (CNCB) (BioCode: BT007725), which is publicly accessible at https://ngdc.cncb.ac.cn/biocode/tool/7725. The web server “*HemaScopeCloud*” is freely accessible at https://hemascope.hiplot.cn/?home=hemascope. The Docker image “*HemaScopeDocker*” can be obtained by pulling from Docker Hub. Detailed tutorials are available online at https://zhenyiwangthu.github.io/HemaScope_Tutorial/.

## CRediT author statement

**Zhenyi Wang:** Conceptualization, Data curation, Formal analysis, Funding acquisition, Investigation, Methodology, Software, Validation, Visualization, Writing ‒ original draft, Writing ‒ review & editing. **Yuxin Miao:** Formal analysis, Investigation, Methodology, Software, Validation, Visualization, Writing ‒ original draft, Writing ‒ review & editing. **Hongjun Li:** Software. **Wenyan Cheng:** Investigation, Validation, Writing ‒ review & editing. **Minglei Shi:** Investigation. **Gang Lv:** Resources. **Yating Zhu:** Software. **Junyi Zhang:** Investigation. **Tingting Tan:** Software. **Jin Gu:** Methodology. **Michael Q. Zhang:** Methodology. **Jianfeng Li:** Resources, Software, Supervision. **Hai Fang:** Funding acquisition, Supervision, Validation, Writing ‒ original draft, Writing ‒ review & editing. **Zhu Chen:** Supervision, Writing ‒ original draft, Writing ‒ review & editing. **Saijuan Chen:** Funding acquisition, Project administration, Supervision, Writing ‒ original draft, Writing ‒ review & editing. All authors have read and approved the final manuscript.

## Competing interests

The authors have declared no competing interests.

## Supplementary Material

qzaf002_Supplementary_Data
