# Supplementary material for: HemaScope: A Tool for Analyzing Single-cell and Spatial Transcriptomics Data of Hematopoietic Cells
Source: Genomics Proteomics Bioinformatics. 2025 Jan 25;23(2):qzaf002. doi: 10.1093/gpbjnl/qzaf002 (PMC12374577; doi:10.1093/gpbjnl/qzaf002)
Supplement: qzaf002_Supplementary_Data [file qzaf002_supplementary_data.zip › supplementary material captions.docx]

**Supplementary material**

**File S1 Details of HemaScope development and data analysis process**

**Figure S1 The cellular hematopoietic hierarchies of normal donors and AML patients**

**A.** The hierarchy of normal BMMCs. Circle colors indicate cell types, and sizes indicate their proportions among all cells. **B.** The hierarchy of normal BMMCs, and both circle colors and sizes indicate their proportions among all cells. **C.** The hierarchy of 87,538 BMMCs from 5 AML patients in the phs000159 dataset. The colors of the circles represent the cell types, and the sizes of the circles represent the proportions of the corresponding cell types among all cells. **D.** The hierarchy of the same dataset as in C. The colors and sizes of the circles represent the proportions of the corresponding cell types among all cells. **E.** The hierarchy of 217,328 BMMCs from 40 AML patients in the GSE130756 dataset. The colors of the circles represent the cell types, and the sizes of the circles represent the proportions of the corresponding cell types among all cells. **F.** The hierarchy of the same dataset as in E. The colors and sizes of the circles represent the proportions of the corresponding cell types among all cells. AML, acute myeloid leukemia; BMMCs, bone marrow mononuclear cells; HSPCs, hematopoietic stem and progenitor cells; HSC/MPP, hematopoietic stem cell/multipotent progenitor; LMPP, lymphoid-primed multipotent progenitor; CLP, common lymphoid progenitor; CMP, common myeloid progenitor; MDP, monocyte-dendritic cell progenitor; GMP, granulocyte-macrophage progenitor; CDP, common dendritic cell progenitor; MEP, megakaryocyte-erythrocyte progenitor; Pro-mono, progenitor monocyte; CD14 mono, classical (CD14^+^CD16^−^) monocyte; CD16 mono, non-classical (CD14^−^CD16^+^) monocyte; Pre-DC, dendritic cell precursor; pDC, plasmacytoid dendritic cell; cDC1, conventional dendritic cell 1; Mo-DC, monocyte-derived dendritic cell; MKP, megakaryocyte progenitor; MK, megakaryocyte; Pro-ery1, proerythroblast 1; Pro-ery2, proerythroblast 2; Ery, erythroblast; Pre-pro-B, B cell progenitor precursor; Early pro-B, early B cell progenitor; Late pro-B, late B cell progenitor; Pre-B, B cell precursor; Immature B, immature B cell; Naive B, naive B cell; Memory B 1, memory B cell 1; Memory B 2, memory B cell 2; CD8 Tnaive, naive CD8^+^ T cell; CD8 Tdpe, KLRG1^+^IL7R^+^ double-positive effector CD8^+^ T; CD8 Tmpe, memory precursor effector CD8^+^ T; CD8 Teff, effector CD8^+^ T cell; CD8 Tex, exhausted CD8^+^ T; CD4 Tnaive, naive CD4^+^ T cell; CD4 Tem, memory CD4^+^ T cell; CD4 Treg, regulatory CD4^+^ T cell; NK, natural killer cell; NK-XCL1, XCL1^+^ natural killer cell.

**Figure S2**  **Intermediate results of HemaScope constructing the human bone marrow cell atlas**

**A.** The distribution of nFeature_RNA, nCount_RNA, and percent.mt. **B.** The UMAP dimensional reduction result with labels for doublets and singlets. **C.** The UMAP dimensional reduction result with labels for datasets, totaling 25 datasets from 20 donors. **D.** The feature plots depicting the expression of marker genes specific to cells in the human bone marrow, with a redder hue indicating higher gene expression levels. **E.** The heatmap depicting the expression levels of marker genes. **F.** The violin plot depicting the expression levels of marker genes. **G.** The dot plot showcasing the top three highly expressed genes in each cluster. Larger dots indicate a higher cell proportion expressing the gene, while a deeper red hue indicates increased average expression within the specific cell type. **H.** The enriched gene network of DEGs in CD10^+^ B cells, *i.e.*, cluster 16 in Figure 2A. **I.** Cell type labels predicted using Seurat and datasets from HematoMap. **J.** Cell type labels predicted using scmap and immunophenotype datasets from abcCellmap. **K.** Cell type labels predicted using Seurat and immunophenotype datasets from abcCellmap. **L.** Cell type labels predicted using scmap and RNACluster datasets from abcCellmap. **M.** Cell type labels predicted using Seurat and RNACluster datasets from abcCellmap. **N.** The *t*-SNE dimensional reduction results with colors representing different cell types. **O.** The PHATE dimensional reduction results with colors representing different cell types. **P.** The stacked area graph illustrating cell type proportions for each donor, with broader areas denoting higher cell type proportions. nFeature_RNA, the number of genes; nCount_RNA, the number of read counts; percent.mt, the percentage of mitochondrial RNA.

**Figure S3**  **Cellular heterogeneity and dynamics in human bone marrow cells**

**A.** The heatmap displaying lineage scores of human bone marrow cells. **B.** The boxplots illustrating Spearman correlation coefficients among single cells within each cell type for every dataset. **C.** GSVA enrichment scores of five clusters in the erythroid lineage-related pathways. **D.** The distribution of cell cycle scores, with G1 phase cells located to the left of the dashed line classified as G0 phase cells. **E.** The proportion of cells in G0, G1, S, and G2M phases in each cell type. **F.** The heatmap of activity scores for classical TF regulons in each cell type. The row names indicate TFs and the number of their target genes. **G.** The RNA velocity calculated by scVelo was mapped onto the layout derived from the DDRTree algorithm in Monocle 2. On the right, the pseudotime calculated by Slingshot was mapped onto the layout. Warmer colors indicate smaller pseudotime values, while cooler colors indicate larger pseudotime values. **H.** The network of cell–cell interaction strength among these 22 cell clusters. **I.** The heatmap illustrating outgoing signaling patterns calculated by CellChat. **J.** The heatmap illustrating incoming signaling patterns calculated by CellChat. **K.** scRNA-seq data of AML mouse bone marrow, annotated with cell type labels provided in the original paper. **L.** Aneuploidy identified by CopyKat in HemaScope through CNV analysis. GMP, granulocyte-macrophage progenitor; HSPC, hematopoietic stem and progenitor cell; MEP, megakaryocyte-erythroid progenitor; Mono pro, monocyte progenitor; PLC, preleukemic and leukemic cell.

**Figure S4**  **The results of microenvironment analysis on IME and IMS samples using HemaScope**

**A.** The spatial distribution of T cell subtypes in the “Hot” sample. **B.** The spatial distribution of T cell subtypes in the “Cold” sample. **C.** The bubble heatmap showing the interaction among four niche clusters in the “Hot” sample. **D.** The spatial distribution of niche clusters in the “Hot” sample. **E.** The heatmap showing the cellular composition of four niche clusters in the IME sample. **F.** The bubble heatmap showing the interaction within four niche clusters in the IME sample. **G.** The boxplot showing the expression levels of *SPP1* and *CD44* in niche clusters 1 and 4 of the IME sample. (*, *P* < 0.05; **, *P* < 0.01; ***, *P* < 0.001; ****, *P* < 0.0001.) **H.** The boxplot showing the expression levels of *SPP1* and *CD44* in tumor and normal areas of the IME sample. **I.** The spatial distribution of expression levels of *CXCL13* and *CXCR5* in the IME sample. **J.** The spatial distribution of CNV states, inferred using CopyKAT in the IMS. **K.** The spatial distribution of expression levels of *SPP1* and *CD44* in the IMS sample. **L.** The spatial distribution of the abundance of CD14^+^ monocytes and CD16^+^ monocytes in the IMS sample. IMS, invasive margin immunosuppressed. CNV, copy number variation.

**Figure S5 The results of analyzing AITL samples using HemaScope**

**A.** The distribution of nUMI, nGene, and spot quality. **B.** Visualization of clusters in space, as well as on the UMAP and the *t*-SNE layouts. **C.** The dot plot of DEGs. **D.** Spatial variable features: the spatial distribution of spatial variable features. **E.** Deconvolution: the spatial distribution of various cell types from the scRNA-seq dataset inferred using cell2location. **F.** Coexistence analysis: the heatmap showing the coexistence scores of T cell subtypes calculated using the correlation-based method. **G.** Niche identification: the distribution of niche clusters identified using the abundance of various cell types. **H.** The dot plot shows the abundance of each cell type in each niche cluster. **I.** Spatial interaction: the bubble heatmap showing the strength and statistical significance of ligand–receptor interactions between the 4th and the 6th niche clusters. **J.** The bubble heatmap shows the strength and statistical significance of pathway interactions between the 4th and 6th niche clusters. AITL, angioimmunoblastic T cell lymphoma; nUMI, the number of unique molecular identifiers; nGene, detected gene counts.

**Figure S6 The results of combining RNA velocity, UMAP, and Slingshot**

**A.** The RNA velocity arrows calculated by scVelo were mapped onto the layout derived from UMAP. **B.** The RNA velocity streams calculated by scVelo were mapped onto the layout derived from UMAP. **C.** The pseudotime calculated by Slingshot was mapped onto the layout derived from UMAP. Warmer colors indicate smaller pseudotime values, while cooler colors indicate larger pseudotime values.

**Figure S7 The results of combining the marker scoring method and cell2location**

**A.** The marker scoring results indicate three subtypes of B cells related to germinal centers. **B.** The cell2location results of the three subtypes in A. **C.** The yellow circles indicate the regions of germinal centers in the tissue pathology image. B_GC_DZ, B cells in germinal center dark zone; B_GC_LZ, B cells in germinal center light zone; B_GC_prePB, B cells in germinal center pre-plasmablast.

**Table S1 Comparison of HemaScope with existing toolkits for scRNA-seq and ST data analysis**

**Table S2 The list of tools used in HemaScope**

**Table S3 Marker genes of human hematopoietic cells**

**Table S4 Marker genes of mouse hematopoietic cells**

**Table S5 Cell cycle-related genes**

**Table S6 Lineage-related genes for humans**

**Table S7 Lineage-related genes for mice**

**Table S8 The list of scRNA-seq and ST datasets used in this study**

**Table S9 The statistical results on cell types, cell counts and percentages**

**Table S10 DEGs of each cluster**

**Table S11 The complete list of TFs and target genes predicted by SCENIC in the dataset of human bone marrow cells**
