# Supplementary material for: HemaScope: A Tool for Analyzing Single-cell and Spatial Transcriptomics Data of Hematopoietic Cells
Source: Genomics Proteomics Bioinformatics. 2025 Jan 25;23(2):qzaf002. doi: 10.1093/gpbjnl/qzaf002 (PMC12374577; doi:10.1093/gpbjnl/qzaf002)
Supplement: qzaf002_Supplementary_Data [file qzaf002_supplementary_data.zip › Table S8.docx]

**Table S8 The list of scRNA-seq and ST datasets used in this study**

| **Accession** | **Source** | **Species** | **Disease/Healthy** | **No. of cells / spots** | **Technology** |
| --- | --- | --- | --- | --- | --- |
| GSE120221 [1] | Bone marrow | *Homo sapiens* | Healthy | 134,474 | 10X Genomics |
| GSE142645 [2] | Bone marrow | *Mus musculus* | *Myc*-driven acute myeloid leukemia (8 weeks after transplant, 2×) | 9149 | 10X Genomics |
| phs000159 [3] | Bone marrow | *Homo sapiens* | Acute myeloid leukemia | 87,538 | 10X Genomics |
| GSE130756 [4] | Bone marrow | *Homo sapiens* | Acute myeloid leukemia | 217,328 | Microwell-seq |
| GSE181304 [5] | Primary central nervous system lymphoma tumor samples | *Homo sapiens* | Primary central nervous system lymphoma | 34,851 | 10X Genomics |
| GSE230207 [5] | Primary central nervous system lymphoma tumor samples | *Homo sapiens* | Primary central nervous system lymphoma | 14,964 | 10X Genomics Visium |
| All raw data [6] was stored in a database in the School of Medicine, Shanghai Jiaotong University. | Lymph node located on angioimmunoblastic T cell lymphoma patient’s neck. | *Homo sapiens* | Angioimmunoblastic T cell lymphoma | 4697 | 10X Genomics Visium |

**References**

[1] Oetjen KA, Lindblad KE, Goswami M, Gui G, Dagur PK, Lai C, et al. Human bone marrow assessment by single-cell RNA sequencing, mass cytometry, and flow cytometry. JCI Insight 2018;3:e124928.

[2] Wu B, Chen X, Pan X, Deng X, Li S, Wang Z, et al. Single-cell transcriptome analyses reveal critical roles of RNA splicing during leukemia progression. PLoS Biol 2023;21:e3002088.

[3] Petti AA, Williams SR, Miller CA, Fiddes IT, Srivatsan SN, Chen DY, et al. A general approach for detecting expressed mutations in AML cells using single cell RNA-sequencing. Nat Commun 2019;10:3660.

[4] Wu J, Xiao Y, Sun J, Sun H, Chen H, Zhu Y, et al. A single-cell survey of cellular hierarchy in acute myeloid leukemia. J Hematol Oncol 2020;13:128.

[5] Xia Y, Sun T, Li G, Li M, Wang D, Su X, et al. Spatial single cell analysis of tumor microenvironment remodeling pattern in primary central nervous system lymphoma. Leukemia 2023;37:1499–510.

[6] Du J, Qiu C, Li WS, Wang B, Han XL, Lin SW, et al. Spatial transcriptomics analysis reveals that CCL17 and CCL22 are robust indicators of a suppressive immune environment in angioimmunoblastic T cell lymphoma (AITL). Front Biosci (Landmark Ed) 2022;27:270.
