# Supplementary material for: HemaScope: A Tool for Analyzing Single-cell and Spatial Transcriptomics Data of Hematopoietic Cells
Source: Genomics Proteomics Bioinformatics. 2025 Jan 25;23(2):qzaf002. doi: 10.1093/gpbjnl/qzaf002 (PMC12374577; doi:10.1093/gpbjnl/qzaf002)
Supplement: qzaf002_Supplementary_Data [file qzaf002_supplementary_data.zip › Table S2.docx]

**Table S2 The list of tools used in HemaScope**

| **Modules** | **Steps** | **Packages and references** | **Methods** |
| --- | --- | --- | --- |
| Module 1:  scRNA-seq pipeline | Quality control & basic analysis for scRNA-seq data | Seurat [1] | Filer cells, select features, normalization, PCA, clustering, remove batch effect, UMAP, *t*-SNE |
|  |  | DoubletFinder [2] | Identify doublets, |
|  | Construct the cell atlas | phateR [3] | Dimensionality reduction |
|  |  | abcCellmap [4] | Cell type annotation |
|  |  | HematoMap (https://github.com/NRCTM-bioinfo/HematoMap) | Cell type reference,  visualization |
|  |  | clusterProfiler [5] | GO enrichment |
|  |  | OpenXGR [6] | Gene network analysis |
|  |  | org.Mm.eg.db (https://bioconductor.org/packages/release/data/annotation/html/org.Mm.eg.db.html) | The database for mouse gene annotation |
|  |  | org.Hs.eg.db (https://bioconductor.org/packages/release/data/annotation/html/org.Hs.eg.db.html) | The database for human gene annotation |
|  | Calculate the cell heterogeneity | scran [7] | Assign cell cycle phase |
|  |  | GSVA [8] | GSVA |
|  |  | limma [9] | Identify differential GSVA terms |
|  | Dynamical analysis | Monocle 2 [10] | Predict cell trajectories |
|  |  | Slingshot [11] | Predict cell trajectories |
|  |  | velocyto [12] | Produce the loom file for scVelo |
|  |  | scVelo [13] | Calculate RNA velocity |
|  |  | SCENIC [14] | TF analysis |
|  |  | arboreto [14] | Gene regulatory network inference |
|  |  | CellChat [15] | Cell–cell interaction analysis |
| Module 2:  ST  pipeline | Data loading | Stereopy [16] | Stereo-seq data loading |
|  | Quality control, dimensionality reduction & clustering for ST data | Seurat [1] | Filter cells, select features, normalization, PCA, clustering, UMAP, *t*-SNE, find DEGs, find SVFs, assign cell cycle scores |
|  | Spatial interaction | COMMOT [17] | Cell–cell interaction analysis in ST |
|  | Spatial CNV analysis | CopyKAT [18] | Infer DNA CNV |
|  | Deconvolution | cell2location [19] | Map scRNA-seq data to spatial transcriptomics |
|  | Niche analysis | COMMOT [17] | Cell–cell interaction analysis in niches |
| Module 3:  GUI & docker & R package | GUI | Shiny (https://cran.r-project.org/web/packages/shiny/index.html) | Develop the GUI |
|  | Docker | Docker [20] | Develop the docker container |
|  | R package | devtools (https://cran.r-project.org/web/packages/devtools/index.html), RStudio [21] | Develop the R package |

*Note*: scRNA-seq, single-cell RNA sequencing; ST, spatial transcriptomics; GUI, graphical user interface; CNV, copy number variation; GSVA, gene set variation analysis; PCA, principal component analysis; UMAP, uniform manifold approximation and projection; *t*-SNE, *t*-distributed stochastic neighbor embedding; GO, gene ontolog; scVelo, a scalable toolkit for RNA velocity analysis in single cells; TF, transcription factor; SVF, spatially variable features.

**References**

[1] Stuart T, Butler A, Hoffman P, Hafemeister C, Papalexi E, Mauck WM 3rd, et al. Comprehensive integration of single-cell data. Cell 2019;177:1888–902.e21.

[2] McGinnis CS, Murrow LM, Gartner ZJ. DoubletFinder: doublet detection in single-cell RNA sequencing data using artificial nearest neighbors. Cell Syst 2019;8:329–37.e4.

[3] Moon KR, van Dijk D, Wang Z, Gigante S, Burkhardt DB, Chen WS, et al. Visualizing structure and transitions in high-dimensional biological data. Nat Biotechnol 2019;37:1482–92.

[4] Xie X, Liu M, Zhang Y, Wang B, Zhu C, Wang C, et al. Single-cell transcriptomic landscape of human blood cells. Natl Sci Rev 2021;8:nwaa180.

[5] Yu G, Wang LG, Han Y, He QY. clusterProfiler: an R package for comparing biological themes among gene clusters. OMICS 2012;16:284–7.

[6] Bao C, Wang S, Jiang L, Fang Z, Zou K, Lin J, et al. OpenXGR: a web-server update for genomic summary data interpretation. Nucleic Acids Res 2023;51:W387–96.

[7] Lun AT, McCarthy DJ, Marioni JC. A step-by-step workflow for low-level analysis of single-cell RNA-seq data with Bioconductor. F1000Res 2016;5:2122.

[8] Hanzelmann S, Castelo R, Guinney J. GSVA: gene set variation analysis for microarray and RNA-seq data. BMC Bioinformatics 2013;14:7.

[9] Ritchie ME, Phipson B, Wu D, Hu Y, Law CW, Shi W, et al. limma powers differential expression analyses for RNA-sequencing and microarray studies. Nucleic Acids Res 2015;43:e47.

[10] Qiu X, Mao Q, Tang Y, Wang L, Chawla R, Pliner HA, et al. Reversed graph embedding resolves complex single-cell trajectories. Nat Methods 2017;14:979–82.

[11] Street K, Risso D, Fletcher RB, Das D, Ngai J, Yosef N, et al. Slingshot: cell lineage and pseudotime inference for single-cell transcriptomics. BMC Genomics 2018;19:477.

[12] La Manno G, Soldatov R, Zeisel A, Braun E, Hochgerner H, Petukhov V, et al. RNA velocity of single cells. Nature 2018;560:494–8.

[13] Bergen V, Lange M, Peidli S, Wolf FA, Theis FJ. Generalizing RNA velocity to transient cell states through dynamical modeling. Nat Biotechnol 2020;38:1408–14.

[14] Aibar S, Gonzalez-Blas CB, Moerman T, Huynh-Thu VA, Imrichova H, Hulselmans G, et al. SCENIC: single-cell regulatory network inference and clustering. Nat Methods 2017;14:1083–6.

[15] Jin S, Guerrero-Juarez CF, Zhang L, Chang I, Ramos R, Kuan CH, et al. Inference and analysis of cell–cell communication using CellChat. Nat Commun 2021;12:1088.

[16] Fang S, Xu M, Cao L, Liu X, Bezulj M, Tan L, et al. Stereopy: modeling comparative and spatiotemporal cellular heterogeneity via multi-sample spatial transcriptomics. bioRxiv 2023; https://doi.org/10.1101/2023.12.04.569485.

[17] Cang Z, Zhao Y, Almet AA, Stabell A, Ramos R, Plikus MV, et al. Screening cell–cell communication in spatial transcriptomics via collective optimal transport. Nat Methods 2023;20:218–28.

[18] Gao R, Bai S, Henderson YC, Lin Y, Schalck A, Yan Y, et al. Delineating copy number and clonal substructure in human tumors from single-cell transcriptomes. Nat Biotechnol 2021;39:599–608.

[19] Kleshchevnikov V, Shmatko A, Dann E, Aivazidis A, King HW, Li T, et al. cell2location maps fine-grained cell types in spatial transcriptomics. Nat Biotechnol 2022;40:661–71.

[20] Merkel D. Docker: lightweight linux containers for consistent development and deployment. Linux J 2014;2014:2.

[21] Racine, J. S. RStudio: a platform-independent IDE for R and Sweave. J Appl Econ 2012;27:167–72.
