# Supplementary material for: HemaScope: A Tool for Analyzing Single-cell and Spatial Transcriptomics Data of Hematopoietic Cells
Source: Genomics Proteomics Bioinformatics. 2025 Jan 25;23(2):qzaf002. doi: 10.1093/gpbjnl/qzaf002 (PMC12374577; doi:10.1093/gpbjnl/qzaf002)
Supplement: qzaf002_Supplementary_Data [file qzaf002_supplementary_data.zip › File S1.docx]

**File S1 Details of HemaScope development and data analysis process**

# HemaScope dissects cellular heterogeneity and dynamics in human bone marrow cells

In this section, we used HemaScope to explore cell type characteristics within the human bone marrow, focusing on cellular heterogeneity and dynamics. Figure S3A presents a heatmap showcasing lineage scores (*LS_i_*) across four cell lineages. The heatmap’s yellow hue indicates a stronger representation of lineage-specific features, while a bluish hue suggesting a weaker representation. As shown in Figure S3A, T cell subtypes exhibit high T/natural killer (T/NK) lineage scores, indicating pronounced features, while other lineages show lower scores. Certain B cell subsets display robust B cell lineage scores, hematopoietic stem/progenitor cells (HSPCs) and monocyte progenitors present elevated HSPC lineage scores, and CD14^+^ monocytes demonstrate high myeloid lineage scores, among other observations. These results indicate that our proposed method in HemaScope for compute lineage scores can accurately estimate the lineage affiliation levels of cells.

To assess cellular heterogeneity, we utilized the Spearman correlation coefficient. Figure S3B showcases box plots illustrating cell similarity within the same cell type across diverse samples. The lower and upper hinges represent the first and third quartiles (the 25th and 75th percentiles), respectively. Notably, natural killer cells (NK cells, cluster 13) exhibit the highest intragroup heterogeneity (lowest homogeneity), while late erythroid progenitors (cluster 9) demonstrate the lowest intragroup heterogeneity (highest homogeneity). Among the five clusters (clusters 6, 9, 10, 15, and 19) of the erythroid lineage, there is similarity in gene expression characteristics, but with differing intragroup heterogeneity. We used the integrated GSVA function in HemaScope to further explore their pathway characteristics, enriching them in erythroid-related pathways depicted in Figure S3C. This revealed significant pathway differences among these clusters.

Moreover, we utilized cell cycle scores (*Score_cycle_*) to predict the cell cycle phases of these cells. Figure S3D shows the distribution of the cell cycle scores, where cells in the left peak demonstrate lower activation within the cell cycle compared to those in the right peak. By employing the *Score_cycle_*, we categorized cell cycle phases (G0, G1, S, and G2M) of these cells. In Figure S3E, proportions of cells in different cell cycle phases per cell type are depicted. Progenitor B cells (cluster 20) exhibit the highest proportions in S and G2M phases, indicating active proliferation, while late and early erythrocytes (clusters 6 and 10) show the highest proportions in G0, signifying cellular quiescence. This observation consistently aligns with the cellular characteristics observed. These results indicate that the *Score_cycle_* in HemaScope is accurate in predicting the cell cycle.

To explore the dynamics features of these cells, we utilized HemaScope to perform transcriptional regulatory network prediction, trajectory analysis, and cell–cell interaction analysis. The heatmap in Figure S3F shows the activity scores of classical hematopoietic-related transcription factor (TF) regulons within each cell, where red signifies higher activity and blue signifies lower activity. The row names of the heatmap indicate the TFs and the numbers of genes in the gene regulatory networks, *e.g.*, “*GATA1* (449g)” means the TF *GATA1* and 449 target genes. Besides the visualization of TF regulons activity, HemaScope also provided a gene list for each TF regulon in the output directory. In Table S11, the table includes 316 TFs and 10,323 target genes in this case study. Specific TF regulons’ high activity characterizes distinct cell types. For example, *GATA3* [1], *RUNX3* [1], *KLF12* [2], *ETS1* [3], *NFATC2* [4], *TBX21* [5], and *EOMES* [5] regulate T cells and NK cells, impacting differentiation, activation, proliferation, immune responses, and cell homeostasis. In monocytes, *MAFB* [6] controls macrophage maturation, while *RARA* [7] regulates homeostasis and oncogenesis. In erythrocytes, *KLF13* [8] regulates the cell cycle, while *KLF1* [9] regulates erythrocyte differentiation and hemoglobin gene expression, respectively. *TAL1* [10] and *GATA1* [11] are crucial in erythroid differentiation, while *NFE2* [12] is vital for megakaryocyte differentiation. Broad functional TFs like *YY1* [13], *MYC* [14], *TCF3* (*E2A*) [15], *EBF1* [16], *PAX5* [16], *BCL11A* [17], *SPIB* [18], and *IRF4* [19] influence gene expression, cell cycle, differentiation, and immune cell functions.

For reconstructing differentiation trajectories, we integrated three different algorithms, including Monocle 2, scVelo, and Slingshot, which are based on different mathematical models to utilize information from different levels of the data, and the results allow mutual validation and complement each other. In Figure S3G, Monocle 2 predicted two trajectories branching from HSPCs and monocyte progenitors, which combine the arrows predicted by scVelo, depicting cell differentiation direction. One to CD14^+^ monocytes, while another branch via CD14^+^ monocytes to CD16^+^ monocytes. The right corner in Figure S3G presents pseudotime calculated by Slingshot, indicating that HSPCs are in the early stage of the cell trajectory. Some CD14^+^ monocytes are in the intermediate stage, while other CD14^+^ monocytes and all CD16^+^ monocytes are in the late stage.

Additionally, we utilized the CellChat module in HemaScope for intercellular interaction analysis through ligand–receptor signaling among cell populations. Figure S3H illustrates interaction strength among these 22 cell clusters, node size reflecting cell counts, edge width indicating interaction strength, and edge color representing the cell cluster expressing ligand. From Figure S3I and J, we can see ligand–receptor signaling heatmaps across 22 cell clusters, with darker shades denoting higher signal intensity. Notably, T cells, B cells, NK cells, and monocyte subpopulations exhibited strong cell–cell interactions. Moreover, integrating CopyKat into HemaScope allowed the prediction of copy number variations (CNVs) from single-cell RNA sequencing (scRNA-seq), identifying aneuploids. Figure S3K displays the acute myeloid leukemia (AML) mouse bone marrow cells [20], labeled in the original source. Figure S3L represents aneuploids identified through CNV prediction, showing overlap with the preleukemic and leukemic cells (PLCs), demonstrating the efficacy of the method.

# The computational details of cellular content of the human bone marrow mononuclear cells determined by scRNA-seq

In Figure 3, the scRNA-seq data were collected from the work of Karolyn and his colleagues [21]. The authors isolated human bone marrow samples through Ficoll density gradient separation, enriching for mononuclear cells. The proportion of T cells within these mononuclear cells was found to be approximately 48.4% using HemaScope, consistent with previous estimations (~ 49.1% of mononuclear cells) from flow cytometry data when accounting for the removal of granulocytes, which constitute the highest proportion of all cells in human bone marrow, approximately ranging from 57% to 86% [22]. And we presented the proportions of 22 cell types within human bone marrow mononuclear cells (BMMCs) in Table S9. These results underscore the effectiveness and robustness of our scRNA-seq data analysis pipeline in accurately identifying cell types within the human bone marrow.

As an example, we clarify the step-by-step process for the calculation of T cell proportion as follows. The proportion of granulocytes is taken as 71.5% (the median between 57% and 86%) [22], and subtracting this from the total bone marrow cells leaves us with 28.5%. The proportion of T lymphocytes is calculated as 14% (the median between 8% and 20%), which is approximately 49.1% when divided by 28.5%. As shown in Figure 3 and Table S9 of our paper, the proportion of T cells within human BMMCs is approximately 48.4%. Therefore, these two proportions are very close to each other. We also compared the proportion of other cell types in Figure 3 of our main text with those in Table 2 of Nombela-Arrieta et al.’s work [22]. The results were consistent, which validated our analytical results.

# The characteristics of tumor boundary of the IMS sample from PCNSL

We also investigated the features of the tumor boundary of invasive margin-excluded (IME) sample from primary central nervous system lymphoma (PCNSL). We found that *SPP1* and *CD44* exhibited high expression at the tumor boundary (Figures S4J and K). Furthermore, deconvolution analysis uncovered a significant presence of CD14^+^ and CD16^+^ monocytes surrounding the tumor (Figure S4L), suggesting the potential existence of TAM*-SPP1* at the tumor boundary in the invasive margin immunosuppressed (IMS) sample, which is similar to the IME sample, as shown in the main text.

# The cell–cell communications between B cells and malignant T cells in AITL

For the angioimmunoblastic T cell lymphoma (AITL) sample, we used HemaScope to calculate normalized unique molecular identifier (UMI) and gene counts for each spot in the sample (Figure S5A). After spot filtration based on predefined thresholds, the data were normalized, dimensionality reduced, clustered, and visualized (Figure S5B). The analysis of differentially expressed gene (DEG) and spatially variable features was conducted (Figures S5C and D). In AITL, the interactions between B cells and malignant T cells, especially malignant T follicular helper cells (Tfh), are very important, and many studies have been conducted on this issue [23,24]. To analyze the interactions between these two cells types in spatial transcriptomics (ST), it is necessary to first locate their spatial positions. In HemaScope, we used cell2location to analyze the spatial distribution of various B cells and T cells in the AITL sample, using three single-cell datasets from human secondary lymphoid organ studies as references [25–27].

As shown in Figure S5E, B cells are primarily aggregated in several areas of the space. Corresponding to the histopathological images, these areas have darker colors, indicating that they are germinal centers, which aligns with the deconvolution results of B cell aggregation. At the same time, the spatial distribution of Tfh cells (Figure S5F), which are closely related to AITL, mainly concentrates in the upper right part of the tissue. Based on their spatial distribution, B cells and T cells are located close to each other in the sample. Therefore, we adopted the method from “NicheAnalysis” in HemaScope, using the proportion of cells obtained from deconvolution as features, to perform K-means clustering of the tissue and obtained 10 different types of microenvironments (Figures S5G and H). By observing the composition of cells in different microenvironments, the 4th microenvironment is mainly composed of T cells, and the 6th microenvironment is mainly composed of B cells. Therefore, we focused on the analysis of these two microenvironments to observe the interaction between B cells and T cells in the space. Then, we analyzed the interactions between the 4th and 6th microenvironments using communication analysis by optimal transport (COMMOT), which accounts for spatial distances when inferring interactions (Figure S5I) and involved pathways (Figure S5J). It is evident that ligand–receptor pairs such as *TNFSF13B–TNFRSF13C* [28], which are related to B cell functional regulation, are significantly expressed. Additionally, ligand–receptor pairs such as *MDK–SDC4* [29], *IL16–CD4* [30], *CXCL16–CXCR6* [31], and *CCL18–ACKR1* [32], which play important roles in the tumor immune microenvironment, are also highly expressed in the interactions among these microenvironments.

# Pitfalls and recommendations for setting cutoffs

In HemaScope, there are numerous customized cutoffs, and setting different cutoffs can impact the data analysis results. The setting of cutoffs is not cell type-specific nor does it have fixed standards but is based on the characteristics of the data. We referred to general practices for scRNA-seq and ST data analysis [33–40], as well as research on hematopoietic cell data analysis [41–45], combined with our understanding of the mathematical principles of various algorithms, to explain the pitfalls and recommendations for setting cutoffs as follows.

In quality control, there are cutoffs for identifying outlier peaks in the number of features, the count depth, and mitochondrial read fraction. We suggest considering these covariates jointly rather than individually. Additionally, we recommend using lenient cutoffs initially and adjusting to stricter cutoffs if low-quality clusters are identified in downstream clustering analysis. For multiple samples, due to variations in sample quality, we suggest setting cutoffs separately for each sample.

In feature selection, dimensionality reduction, and visualization, it is necessary to set the number of selected features, dimensions for dimensionality reduction, and dimensions of the visualization space. Feature selection is typically based on the principle of maximizing “information” to select highly variable genes (HVGs), with the cutoff usually ranging from 1000 to 5000. The downstream analysis results are robust to the choice of this cutoff [46]. In dimensionality reduction, the underlying assumption is that high-dimensional biological data can be embedded on a low-dimensional manifold. This is typically achieved using linear or nonlinear methods to project data with selected features into a low-dimensional space, and primarily aimed at addressing the curse of dimensionality. The selection of cutoffs involves many data-driven methods, such as using elbow plots to determine the cutoffs of dimensions for principal component analysis (PCA) [41]. In visualization, we also need to specify the number of dimensions for visualizing the data. Due to the fact that most humans can only comprehend 3-dimensional space or fewer through visual perception, this cutoff is typically set to 1, 2, or 3 dimensions.

In clustering, we recommend using different cutoffs for the resolution to identify clusters of cells, aiding in the discovery of potential subpopulations within known cell groups.

# The visual representation of the cellular hematopoietic hierarchy for cells in human bone marrow

Exploring the cellular hematopoietic hierarchy is a critical task in hematopoietic cell research. Various visualization methods are instrumental in addressing this task. PCA is a linear dimensionality reduction technique, which is suitable for visualizing data with simple structures or as a preliminary process for downstream analysis to mitigate the effects of the curse of dimensionality. The *t*-distributed Stochastic Neighbor Embedding (*t*-SNE) focuses on the local structure of the data, making it more suitable for distinguishing cell clusters, while Potential of Heat-diffusion for Affinity-based Trajectory Embedding (PHATE) emphasizes preserving the continuity of the data, making it suitable for observing differentiation trajectories. Uniform Manifold Approximation and Projection (UMAP) balances both local and global structures, maintaining a balance between distinguishing cell clusters and preserving differentiation trajectories. Integrating PCA, UMAP, *t*-SNE, and PHATE within HemaScope can help comprehensively reveal low-dimensional characteristics. Recently, Dai et al. proposed a hematopoietic cell-specific R package named HematoMap (https://github.com/NRCTM-bioinfo/HematoMap), which provides a visual representation of the cellular hematopoietic hierarchy for cells in human bone marrow. We integrated its visualization approach into HemaScope. As shown in Figure S1A, the tree-like structure illustrates the hierarchy of normal BMMCs. The colors of the circles represent the cell types, while the sizes of the circles represent the proportions of the corresponding cell types among all cells. To more intuitively display the proportions, the color of the circles can also be shaded according to the proportions (Figure S1B). For AML patients, the cellular hierarchy of BMMCs shows a significant shift. We collected scRNA-seq data of 87,538 bone marrow cells from 5 AML patients (Figure S1C and D) in the work of Petti et al. [47] and 217,328 bone marrow cells from 40 AML patients (Figure S1E and F) in the work of Wu et al. [48] via the ABC portal database [49], respectively. As shown in Figure S1C–F, the myeloid cells significantly increase while lymphoid cells significantly decrease in AML patients. Meanwhile, we also observed that the differentiation of HSPCs was blocked at different stages in different AML patients.

# Technical details in cell type annotation process

In the process of cell type annotation, we integrated seven methods to improve its accuracy. The first method involves profiling hematopoietic cell-specific marker gene expression. We collected marker genes of human and mouse hematopoietic cells from references [42,44,50–52], CellMarker 2.0 [53] and online data resources (https://github.com/NRCTM-bioinfo/HematoMap). In Tables S3 and S4, we list the marker genes for 140 populations of human hematopoietic cells and 118 populations of mouse hematopoietic cells. To integrate the advantages of various visualization techniques for presenting marker gene expression profiles, HemaScope provides three visualization methods including dot plots, violin plots, and heatmaps. These marker gene expression profiles form the foundational for cell type annotation.

The second method is differential gene expression analysis. We utilized the Wilcoxon rank-sum test with Bonferroni correction in Seurat [54] to identify sets of genes that are significantly highly expressed within each cell cluster. To filter these genes, we selected genes expressed in at least 25% of cells within each cluster by setting “min.pct” to 0.25, with the average expression in that cluster showing at least an X-fold difference (on a log_2_ scale) compared to the average expression in all other clusters by setting “logfc.threshold” to 0.25. These significantly highly expressed genes within each cluster can serve as a supplement to well-known marker genes for identifying cell types and may also reveal new characteristics of these cells.

The third method is Gene Ontology (GO) enrichment. We utilized the “enrichGO” function in clusterProfiler [55] with a false discovery rate (FDR) correction to enrich the significantly highly expressed gene sets of each cluster into biological processes. The top 30 enriched results (or all results if fewer than 30 enrichments were found) were visualized using a bar plot. Additionally, all enrichment results were output as a table in comma-separated values (CSV) format. These GO enrichment results reflect the characteristics of these cells from the perspective of biological processes, enhancing the accuracy of cell type annotation and providing insight into the potential biological processes occurring within these cells, thereby contributing to a more comprehensive understanding of the data.

The fourth method is Kyoto Encyclopedia of Genes and Genomes (KEGG) pathway enrichment. Similar to GO enrichment, we utilized the “enrichKEGG” function in clusterProfiler [55] with FDR to enrich the significantly highly expressed gene sets of each cluster into KEGG pathways. Then, we visualized and output the KEGG enrichment results using the same strategies as those used for GO enrichment. These results reflect the characteristics of the cells from a pathway perspective.

The fifth method is gene network analysis. We used the gene network analyzer in OpenXGR [56] to identify gene subnetworks from the significantly highly expressed gene sets of each cluster. The analyzer leverages the knowledge of functional networks from STRING [57] database and pathways from KEGG database [58]. Thus, we integrated this tool to explore the cells from the gene network level.

The sixth method is based on ChatGPT (v.3.5) from OpenAI. Hou and Ji demonstrated that the large language model can enhance the accuracy of cell type annotation by using marker gene information [59]. We integrated the “gptcelltype” function from GPTCelltype [59] into our toolkit. It takes the highly expressed genes or marker genes and the tissue name as inputs. Then it outputs a prompt for ChatGPT. For example, we input the marker genes including “*CD4*, *CCR7*, *SELL*, *LEF1*” and the tissue name, “human bone marrow”. It outputs the prompt: “Identify cell types of human bone marrow cells using the following markers separately for each row. Only provide the cell type name. Do not show numbers before the name. Some can be a mixture of multiple cell types.\n *CD4*, *CCR7*, *SELL*, *LEF1*”. Then we manually input this prompt to ChatGPT (v.3.5) via its web version. It returned “Naive T cells”. In this way, ChatGPT serves as an assistant to annotate cell types.

The seventh method is based on label transfer. This strategy uses annotated scRNA-seq datasets with cell type labels as references and employs data integration algorithms, such as “anchor-based” integration workflow in Seurat [54] and the “BLAST-similar” method for projecting cells in scmap [60], to map the dataset requiring annotation onto the reference data, thereby achieving the matching shared cell types and states. We collected well annotated scRNA-seq datasets from publicly available data resources and employed Seurat to annotate input datasets based on these data. Seurat not only provides predicted cell type labels but also assigns reliability scores for each cell’s association with different cell types. HemaScope utilizes these scores to color the UMAP layout and provides the figures to users. Meanwhile, users also can input their own annotated datasets as references. Additionally, we also integrated abcCellmap [61], which serves as a blood cell-specific annotation tool, into our toolkit. The abcCellmap uses Seurat and scmap as data integration algorithms. It uses a reference dataset labeled by 43 cell clusters derived from unsupervised clustering and another reference dataset labeled by 32 immunophenotypic cell types. The aforementioned methods were integrated into our toolkit to transfer cell type labels from well-annotated reference datasets to the input data. In HemaScope, we have configured an adjustable parameter named “Step4_Use_Which_Labels” in the function “scRNASeq_10x_pipeline” to select the source of cell type labels. Specifically, “HematoMap” indicates the use of the Seurat method to integrate reference data and input data, utilizing reference data from “HematoMap”. “abcCellmap.1” indicates the use of scmap and 32 immunophenotypic cell types reference data, “abcCellmap.2” indicates the use of Seurat and 32 immunophenotypic cell types reference data, “abcCellmap.3” indicates the use of scmap and 43 cell clusters reference data, “abcCellmap.4” indicates the use of Seurat and 43 cell clusters reference data, and “clustering” indicates the use of labels obtained solely from unsupervised clustering without the aforementioned strategies.

Cell type annotation for scRNA-seq remains an unresolved problem, as no existing method can achieve 100% accuracy across all datasets. Therefore, in HemaScope, we have integrated the aforementioned seven methods to allow users to combine the results from these methods, thereby enhancing the accuracy.

# Cell cycle score designed for hematopoietic cells

An emerging theme in hematopoietic stem cell (HSCs) and leukemia research is that transcriptional regulators orchestrate the equilibrium between cell proliferation and quiescence within the HSC pool by modulating the quantity and frequency of cells transitioning through the cell cycle [62,63]. In this paper, we referred to the work of Dong et al. [42] to further introduce a parameter named cell cycle score (*Score_cycle_*), combined with the data-driven machine learning method called scran [64] to classify single cells into G0, G1, S, and G2M phases based on cell cycle-related transcriptional profiling of individual hematopoietic cells.

The biological significance of *Score_cycle_* is grounded in two biological facts. The first one [65,66] is that specific gene expression changes in protein abundance, isoform expression, and phosphorylation occur during various phases of the cell cycle. The second one [66–68] is that cells in the cell cycle exhibit higher expression levels of cell cycle-related genes compared to cells not in the cell cycle, which leads to the expression levels of cell cycle-related genes to present a bimodal distribution. Based on these facts, we designed a strategy to classify cells into different cell cycle phases as follows. First, we utilized the “cyclone” function in scran [64] package to classify cells in G1, S, and G2M phases based on expression levels of specific genes in each cell cycle phase and pre-trained classifiers. Second, to further distinguish cells in the G0 phase, we assigned the G1 phase cells from the left peak of the *Score_cycle_* bimodal distribution to the G0 phase, as these cells exhibited lower translation activity during the G1 phase and were more likely to be in the G0 phase.

Specifically, the bimodal distribution of the cell cycle score was referenced from a paper [68] by single-cell experts at Aviv Regev’s lab at the Broad Institute. The lower transcriptional activity of G1/G0 phase cells compared to S/G2/M phase cells was referenced from a paper [66] by hematology experts at Bo Torben Porse’s lab at the University of Copenhagen and Ido Amit’s lab at the Weizmann Institute of Science. The strategy of combining the cell cycle score and scran to classify the cell cycle was referenced from a paper [42] by hematology experts at Tao Cheng’s lab at the Chinese Academy of Medical Sciences and Peking Union Medical College.

In terms of computational details, the cell cycle status of each cell is predicted using the “cyclone” method from scran [64], distinguishing among G1, S, and G2M phases. Cell cycle scores are derived from gene sets active during the cell cycle, following this formula:

$$Score_{cycle}=\frac{\sum_{i=1}^{n} D(g_{i})}{n},g_{i}\in G_{cycle}$$

Here, *G_cycle_* represents the cell cycle-related genes (Table S5), and *D*(*g_i_*) represents the normalized expression value of gene *g_i_* within the “data” slot in the Seurat object. Meanwhile, *n* stands for the total number of genes present within *G_cycle_*. Higher computed values signify greater cell cycle activation, while lower ones indicate reduced activation. In the presence of a bimodal *Score_cycle_* distribution, cells in the left peak are considered relatively quiescent, while those in the right peak are viewed as relatively active. Cells with lower scores in the G1 phase are likely to be representing the G0 phase cells, inferred from observed bimodal distributions.

# Lineage score designed for hematopoietic cells

In this work, we designed a parameter called lineage score (*LS_i_*) which can quantitatively measure the affiliation levels of individual cells to various lineages within the hematopoietic hierarchy based on the expression levels of gene sets associated with each hematopoietic lineage. *LS_i_* not only helps to more accurately identify cell types but also indicates the potential of HSPCs to differentiate into different lineages. Furthermore, in leukemia research, using *LS_i_* to measure the lineage scores of leukemia cells helps accurately locate the position where cell blockage occurs in the hematopoietic hierarchy.

In terms of computational details, distinct lineage related gene sets for humans (*Homo sapiens*, *hsa*, Table S6) [53] and mice (*Mus musculus*, *mmu*, Table S7) [66] delineating various hematopoietic lineages are utilized, respectively. Table S6 lists HSPCs, myeloid cells, B cells, T/NK cells for humans. Table S7 lists these gene sets, including HSCs, multipotent progenitor (MPPs), erythroid cells, lymphoid cells, and myeloid cells for mice. The lineage score *LS_i_* of cell *i* with gene set *G* is defined as follows:

$$LS_{i}=\frac{\sum_{j} g_{i,j}}{\#\{j\left|j\in G\right.\}},j\in G$$

where *g_i,j_* denotes the UMI count of gene *j* in cell *i*, and *#* represents the number of genes in gene set *G*. Subsequently, the lineage scores per cell are normalized through mean subtraction and division by standard deviation. Violin plots and heatmaps visualize gene expression profiles and lineage scores.

# Hematopoietic cell-specific gene sets

In the computation of lineage scores, cell cycle scores, and cell type identification for hematopoietic cells, the utilization of hematopoietic cell-specific gene sets is essential. We collected 71 genes for four lineages of humans (Table S6) and 48 genes for five hematopoietic cell lineages of mice (Table S7) from references [53,66]. To calculate cell cycle scores, we collected 97 cell cycle-related genes in hematopoietic cells (Table S5) [42]. For cell type identification, we collected 588 marker genes of 140 cell populations for humans and 1622 marker genes of 118 cell populations for mice [53,66] (see Tables S3 and S4).

# Comparing Monocle 2, scVelo, and Slingshot

From an algorithmic perspective, Monocle 2, scVelo, and Slingshot each leverage distinct principles to analyze cellular trajectories. Monocle 2 utilizes the continuity of data points in high-dimensional space to construct a tree-like structure of the data, which helps identify bifurcated paths in cellular trajectories. However, it does not infer the specific directions within these trajectories. In contrast, scVelo leverages information about splicing and unsplicing from scRNA-seq data to predict both the differentiation direction and rate of each cell at the subsequent time point. Slingshot utilizes the principal curve of data points in PCA space to calculate pseudotime, which provides an intuitive reflection of the sequential order of data points within a high-dimensional space. Among these methods, scVelo, based on RNA velocity, can provide the differentiation direction and rate for each cell. To facilitate readers’ understanding, we provide a brief explanation of RNA velocity here. The fundamental assumption of RNA velocity posits that the ratio of unspliced to spliced messenger RNA (mRNA) abundance increases for genes expected to have increasing expression levels; vice versa. The RNA velocity model establishes a system of differential equations describing mRNA abundance over time, aiming to predict the differentiation direction and rate of each cell at the next time point. When integrated with visualization methods, such as UMAP, *t*-SNE, and so on, it enables detailed studies of cell trajectories at a single-cell resolution.

From the perspective of data analysis practice, Monocle 2 employs the reversed graph embedding method to reconstruct a tree-like structure of the data. Therefore, Monocle 2 can discover bifurcated paths in cellular trajectories but cannot infer the directions within the cellular trajectories. scVelo can predict the differentiation direction and rate of each cell at the subsequent time point. The computation process of Slingshot is the simplest among the three. It uses a minimum spanning tree in PCA space to derive the principal curve of the data. Therefore, the pseudotime calculated by Slingshot can intuitively reflect the sequential order of data points in high-dimensional space. There is a “no free lunch theorem” [69] in machine learning that emphasizes the fact that there is no universal algorithm that outperforms all others across all possible problems. In essence, while certain algorithms may excel in one scenario, they may perform poorly in another. To visually demonstrate this, we projected the RNA velocity calculated by scVelo and the pseudotime calculated by Slingshot onto the layout derived from the DDRtree algorithm in Monocle 2 (Figure S3G). Furthermore, we combined the RNA velocity arrows, RNA velocity streams, and pseudotime among HSPC, monocyte progenitors, CD14^+^ monocytes, and CD16^+^ monocytes predicted by scVelo with the UMAP layout in Figure S6A–C. Compared with Figure S3G, we can see that the UMAP layout tends to obtain clusters, but Monocle 2 layout tends to obtain bifurcated paths. Thus, we integrate various algorithms that can mutually validate and complement each other in HemaScope.

# The computational details of analyzing the spatial distribution of cell types using cell2location and marker gene scoring

HemaScope implemented cell2location [70] and marker gene scoring to comprehensively analyze the spatial distribution of cell types in samples generated by the 10X Visium platform. cell2location [70] is a Bayesian model-based approach that integrates single-cell transcriptomic reference data with ST data to infer the distribution of each cell type in space, thereby converting spatially mixed signals into information about the composition of cells at different locations. It was benchmarked as the “top-performing” method in cell type deconvolution, effectively delineating the spatial distribution of different cell types [71]. We employed cell2location as the core method to map reference scRNA-seq data, derived from either HematoMap (https://github.com/NRCTM-bioinfo/HematoMap) or three human secondary lymphoid organ studies [25–27] as default, onto the ST data. This process facilitates the inference of the abundance distribution of diverse cell types from the mixed signals present in 10X Visium data.

Meanwhile, we incorporated a process into our pipeline to score marker genes for each cell type and visualize them as a complement to the deconvolution results from cell2location. The marker gene scoring method used the expression of marker genes in tissues for each cell type to determine their spatial distributions. Each marker for a cell type is derived from the DEG analysis conducted on the reference single-cell datasets mentioned above. The DEG analysis employs the “scanpy.tl.rank_genes_groups” method of SCANPY [72]. We select the top 50 genes with the highest scores as markers for each cell type. Scoring on ST data is performed using “AddModuleScore” function in Seurat [54]. As shown in Figure S7A, the marker scoring results indicate that nearly all B cell subtypes cluster in a few regions within the tissue, especially those subtypes related to germinal centers (B_GC_DZ, B_GC_LZ, and B_GC_prePB). However, it is not obvious in the results from cell2location (Figure S7B). According to the tissue pathology image (Figure S7C), there are regions with a dark color (within yellow circles), which indicate a high probability of being germinal centers. Consequently, the marker scoring results could significantly enhance the accuracy of cell type annotations.

# The definition of the coexistence score

The coexistence score based on Wasserstein distance for cell types *i* and *j* is defined as follows:

$Score_{i,j}=\frac{1}{1+d_{i,j}}$,

where *d_i,j_* is the Wasserstein distance between cell types *i* and *j*. A high *Score_i,j_* signifies robust global coexistence between cell types *i* and *j*, enhancing the reliability of interaction analysis.

# Balance the simplicity and flexibility of HemaScope

To balance the simplicity and flexibility of HemaScope, we specifically incorporated the following designs: (1) adding “select” or “skip” functionalities in each step, allowing users to customize the analysis tasks in the pipeline freely; (2) saving all intermediate results of each step as completely as possible, allowing users to conveniently access them for additional analyses; (3) providing comprehensive parameters for users to customize, along with default values based on our experience and experiments; (4) developing both a user-friendly interactive interface and an R language command-line mode, allowing researchers without programming experience to analyze data in a code-free manner, while also enabling researchers with programming experience to easily perform secondary development using the open-source R language functions to meet their specific research needs; (5) developing an online *HemaScopeCloud* data analysis platform, which is freely available to researchers in the scientific community for analyzing their own data. Additionally, we will continuously improve and upgrade our toolkit based on user feedback.

#

# References

[1] Yagi R, Junttila IS, Wei G, Urban JF Jr, Zhao K, Paul WE, et al. The transcription factor GATA3 actively represses RUNX3 protein-regulated production of interferon-gamma. Immunity 2010;32:507–17.

[2] Lam VC, Folkersen L, Aguilar OA, Lanier LL. KLF12 regulates mouse NK cell proliferation. J Immunol 2019;203:981–9.

[3] Taveirne S, Wahlen S, Van Loocke W, Kiekens L, Persyn E, Van Ammel E, et al. The transcription factor ETS1 is an important regulator of human NK cell development and terminal differentiation. Blood 2020;136:288–98.

[4] Macian F. NFAT proteins: key regulators of T-cell development and function. Nat Rev Immunol 2005;5:472–84.

[5] Kiekens L, Van Loocke W, Taveirne S, Wahlen S, Persyn E, Van Ammel E, et al. T-BET and EOMES accelerate and enhance functional differentiation of human natural killer cells. Front Immunol 2021;12:732511.

[6] Hamada M, Tsunakawa Y, Jeon H, Yadav MK, Takahashi S. Role of MafB in macrophages. Exp Anim 2020;69:1–10.

[7] Duong V, Rochette-Egly C. The molecular physiology of nuclear retinoic acid receptors. From health to disease. Biochim Biophys Acta 2011;1812:1023–31.

[8] Zhang W, Hong S, Maniar KP, Cheng S, Jie C, Rademaker AW, et al. KLF13 regulates the differentiation-dependent human papillomavirus life cycle in keratinocytes through STAT5 and IL-8. Oncogene 2016;35:5565–75.

[9] Perkins A, Xu X, Higgs DR, Patrinos GP, Arnaud L, Bieker JJ, et al. Krüppeling erythropoiesis: an unexpected broad spectrum of human red blood cell disorders due to KLF1 variants. Blood 2016;127:1856–62.

[10] Vagapova ER, Spirin PV, Lebedev TD, Prassolov VS. The role of TAL1 in hematopoiesis and leukemogenesis. Acta Naturae 2018;10:15–23.

[11] Briegel K, Bartunek P, Stengl G, Lim KC, Beug H, Engel JD, et al. Regulation and function of transcription factor GATA-1 during red blood cell differentiation. Development 1996;122:3839–50.

[12] Fujita R, Takayama-Tsujimoto M, Satoh H, Gutiérrez L, Aburatani H, Fujii S, et al. NF-E2 p45 is important for establishing normal function of platelets. Mol Cell Biol 2013;33:2659–70.

[13] Rizkallah R, Hurt MM. Regulation of the transcription factor YY1 in mitosis through phosphorylation of its DNA-binding domain. Mol Biol Cell 2009;20:4766–76.

[14] Delgado MD, Leon J. *Myc* roles in hematopoiesis and leukemia. Genes Cancer 2010;1:605–16.

[15] Laidlaw BJ, Cyster JG. Transcriptional regulation of memory B cell differentiation. Nat Rev Immunol 2021;21:209–20.

[16] Somasundaram R, Jensen CT, Tingvall-Gustafsson J, Ahsberg J, Okuyama K, Prasad M, et al. EBF1 and PAX5 control pro-B cell expansion via opposing regulation of the *Myc* gene. Blood 2021;137:3037–49.

[17] Psatha N, Reik A, Phelps S, Zhou Y, Dalas D, Yannaki E, et al. Disruption of the BCL11A erythroid enhancer reactivates fetal hemoglobin in erythroid cells of patients with beta-thalassemia major. Mol Ther Methods Clin Dev 2018;10:313−26.

[18] Takagi Y, Shimada K, Shimada S, Sakamoto A, Naoe T, Nakamura S, et al. SPIB is a novel prognostic factor in diffuse large B-cell lymphoma that mediates apoptosis via the PI3K–AKT pathway. Cancer Sci 2016;107:1270–80.

[19] Shukla V, Lu R. IRF4 and IRF8: governing the virtues of B lymphocytes. Front Biol (Beijing) 2014;9:269–82.

[20] Wu B, Chen X, Pan X, Deng X, Li S, Wang Z, et al. Single-cell transcriptome analyses reveal critical roles of RNA splicing during leukemia progression. PLoS Biol 2023;21:e3002088.

[21] Oetjen KA, Lindblad KE, Goswami M, Gui G, Dagur PK, Lai C, et al. Human bone marrow assessment by single-cell RNA sequencing, mass cytometry, and flow cytometry. JCI Insight 2018;3:e124928.

[22] Nombela-Arrieta C, Manz MG. Quantification and three-dimensional microanatomical organization of the bone marrow. Blood Adv 2017;1:407–16.

[23] Leca J, Lemonnier F, Meydan C, Foox J, El Ghamrasni S, Mboumba DL, et al. IDH2 and TET2 mutations synergize to modulate T follicular helper cell functional interaction with the AITL microenvironment. Cancer Cell 2023;41:323–39.e10.

[24] Witalis M, Chang J, Zhong MC, Bouklouch Y, Panneton V, Li J, et al. Progression of AITL-like tumors in mice is driven by Tfh signature proteins and T–B cross talk. Blood Adv 2020;4:868–79.

[25] James KR, Gomes T, Elmentaite R, Kumar N, Gulliver EL, King HW, et al. Distinct microbial and immune niches of the human colon. Nat Immunol 2020;21:343–53.

[26] Park JE, Botting RA, Dominguez Conde C, Popescu DM, Lavaert M, Kunz DJ, et al. A cell atlas of human thymic development defines T cell repertoire formation. Science 2020;367:eaay3224.

[27] King HW, Orban N, Riches JC, Clear AJ, Warnes G, Teichmann SA, et al. Single-cell analysis of human B cell maturation predicts how antibody class switching shapes selection dynamics. Sci Immunol 2021;6:eabe6291.

[28] de Mattos Barbosa MG, Lefferts AR, Huynh D, Liu H, Zhang Y, Fu B, et al. *TNFRSF13B* genotypes control immune-mediated pathology by regulating the functions of innate B cells. JCI Insight 2021;6:e150483.

[29] Hashimoto M, Kojima Y, Sakamoto T, Ozato Y, Nakano Y, Abe T, et al. Spatial and single-cell colocalisation analysis reveals MDK-mediated immunosuppressive environment with regulatory T cells in colorectal carcinogenesis. EBioMedicine 2024;103:105102.

[30] Mo Z, Liu D, Chen Y, Luo J, Li W, Liu J, et al. Single-cell transcriptomics reveals the role of macrophage-naive CD4^+^ T cell interaction in the immunosuppressive microenvironment of primary liver carcinoma. J Transl Med 2022;20:466.

[31] Lesch S, Blumenberg V, Stoiber S, Gottschlich A, Ogonek J, Cadilha BL, et al. T cells armed with C-X-C chemokine receptor type 6 enhance adoptive cell therapy for pancreatic tumours. Nat Biomed Eng 2021;5:1246–60.

[32] Oh K, Yoo YJ, Torre-Healy LA, Rao M, Fassler D, Wang P, et al. Coordinated single-cell tumor microenvironment dynamics reinforce pancreatic cancer subtype. Nat Commun 2023;14:5226.

[33] Luecken MD, Theis FJ. Current best practices in single-cell RNA-seq analysis: a tutorial. Mol Syst Biol 2019;15:e8746.

[34] Heumos L, Schaar AC, Lance C, Litinetskaya A, Drost F, Zappia L, et al. Best practices for single-cell analysis across modalities. Nat Rev Genet 2023;24:550–72.

[35] Hao Y, Stuart T, Kowalski MH, Choudhary S, Hoffman P, Hartman A, et al. Dictionary learning for integrative, multimodal and scalable single-cell analysis. Nat Biotechnol 2024;42:293–304.

[36] Yuan Z, Zhao F, Lin S, Zhao Y, Yao J, Cui Y, et al. Benchmarking spatial clustering methods with spatially resolved transcriptomics data. Nat Methods 2024;21:712–22.

[37] Song Y, Miao Z, Brazma A, Papatheodorou I. Benchmarking strategies for cross-species integration of single-cell RNA sequencing data. Nat Commun 2023;14:6495.

[38] Pullin JM, McCarthy DJ. A comparison of marker gene selection methods for single-cell RNA sequencing data. Genome Biol 2024;25:56.

[39] Fang S, Chen B, Zhang Y, Sun H, Liu L, Liu S, et al. Computational approaches and challenges in spatial transcriptomics. Genomics Proteomics Bioinformatics 2023;21:24–47.

[40] Marconato L, Palla G, Yamauchi KA, Virshup I, Heidari E, Treis T, et al. SpatialData: an open and universal data framework for spatial omics. Nat Methods 2024; https://doi.org/10.1038/s41592-024-02212-x.

[41] Wang Z, Zhong Y, Ye Z, Zeng L, Chen Y, Shi M, et al. MarkovHC: Markov hierarchical clustering for the topological structure of high-dimensional single-cell omics data with transition pathway and critical point detection. Nucleic Acids Res 2022;50:46–56.

[42] Dong F, Hao S, Zhang S, Zhu C, Cheng H, Yang Z, et al. Differentiation of transplanted haematopoietic stem cells tracked by single-cell transcriptomic analysis. Nat Cell Biol 2020;22:630–9.

[43] Cortes-Lopez M, Chamely P, Hawkins AG, Stanley RF, Swett AD, Ganesan S, et al. Single-cell multi-omics defines the cell-type-specific impact of splicing aberrations in human hematopoietic clonal outgrowths. Cell Stem Cell 2023;30:1262–81.e8.

[44] Zhang X, Song B, Carlino MJ, Li G, Ferchen K, Chen M, et al. An immunophenotype-coupled transcriptomic atlas of human hematopoietic progenitors. Nat Immunol 2024;25:703–15.

[45] Mumme H, Thomas BE, Bhasin SS, Krishnan U, Dwivedi B, Perumalla P, et al. Single-cell analysis reveals altered tumor microenvironments of relapse- and remission-associated pediatric acute myeloid leukemia. Nat Commun 2023;14:6209.

[46] Klein AM, Mazutis L, Akartuna I, Tallapragada N, Veres A, Li V, et al. Droplet barcoding for single-cell transcriptomics applied to embryonic stem cells. Cell 2015;161:1187–201.

[47] Petti AA, Williams SR, Miller CA, Fiddes IT, Srivatsan SN, Chen DY, et al. A general approach for detecting expressed mutations in AML cells using single cell RNA-sequencing. Nat Commun 2019;10:3660.

[48] Wu J, Xiao Y, Sun J, Sun H, Chen H, Zhu Y, et al. A single-cell survey of cellular hierarchy in acute myeloid leukemia. J Hematol Oncol 2020;13:128.

[49] Gao X, Hong F, Hu Z, Zhang Z, Lei Y, Li X, et al. ABC portal: a single-cell database and web server for blood cells. Nucleic Acids Res 2023;51:D792–804.

[50] Giladi A, Paul F, Herzog Y, Lubling Y, Weiner A, Yofe I, et al. Single-cell characterization of haematopoietic progenitors and their trajectories in homeostasis and perturbed haematopoiesis. Nat Cell Biol 2018;20:836–46.

[51] Regev A, Teichmann SA, Lander ES, Amit I, Benoist C, Birney E, et al. The Human Cell Atlas. Elife 2017;6:e27041.

[52] Paul F, Arkin Y, Giladi A, Jaitin DA, Kenigsberg E, Keren-Shaul H, et al. Transcriptional heterogeneity and lineage commitment in myeloid progenitors. Cell 2016;164:325.

[53] Hu C, Li T, Xu Y, Zhang X, Li F, Bai J, et al. CellMarker 2.0: an updated database of manually curated cell markers in human/mouse and web tools based on scRNA-seq data. Nucleic Acids Res 2023;51:D870–6.

[54] Stuart T, Butler A, Hoffman P, Hafemeister C, Papalexi E, Mauck WM 3rd, et al. Comprehensive integration of single-cell data. Cell 2019;177:1888–902.e21.

[55] Yu G, Wang LG, Han Y, He QY. clusterProfiler: an R package for comparing biological themes among gene clusters. OMICS 2012;16:284–7.

[56] Bao C, Wang S, Jiang L, Fang Z, Zou K, Lin J, et al. OpenXGR: a web-server update for genomic summary data interpretation. Nucleic Acids Res 2023;51:W387–96.

[57] Szklarczyk D, Kirsch R, Koutrouli M, Nastou K, Mehryary F, Hachilif R, et al. The STRING database in 2023: protein–protein association networks and functional enrichment analyses for any sequenced genome of interest. Nucleic Acids Res 2023;51:D638–46.

[58] Kanehisa M, Furumichi M, Sato Y, Kawashima M, Ishiguro-Watanabe M. KEGG for taxonomy-based analysis of pathways and genomes. Nucleic Acids Res 2023;51:D587–92.

[59] Hou W, Ji Z. Assessing GPT-4 for cell type annotation in single-cell RNA-seq analysis. Nat Methods 2024;21:1462–65.

[60] Kiselev VY, Yiu A, Hemberg M. scmap: projection of single-cell RNA-seq data across data sets. Nat Methods 2018;15:359–62.

[61] Xie X, Liu M, Zhang Y, Wang B, Zhu C, Wang C, et al. Single-cell transcriptomic landscape of human blood cells. Natl Sci Rev 2021;8:nwaa180.

[62] Jude CD, Gaudet JJ, Speck NA, Ernst P. Leukemia and hematopoietic stem cells: balancing proliferation and quiescence. Cell Cycle 2008;7:586–91.

[63] Ling VY, Straube J, Godfrey W, Haldar R, Janardhanan Y, Cooper L, et al. Targeting cell cycle and apoptosis to overcome chemotherapy resistance in acute myeloid leukemia. Leukemia 2023;37:143–53.

[64] Lun AT, McCarthy DJ, Marioni JC. A step-by-step workflow for low-level analysis of single-cell RNA-seq data with Bioconductor. F1000Res 2016;5:2122.

[65] Ly T, Ahmad Y, Shlien A, Soroka D, Mills A, Emanuele MJ, et al. A proteomic chronology of gene expression through the cell cycle in human myeloid leukemia cells. Elife 2014;3:e01630.

[66] Lauridsen FKB, Jensen TL, Rapin N, Aslan D, Wilhelmson AS, Pundhir S, et al. Differences in cell cycle status underlie transcriptional heterogeneity in the HSC compartment. Cell Rep 2018;24:766–80.

[67] Liu Z, Lou H, Xie K, Wang H, Chen N, Aparicio OM, et al. Reconstructing cell cycle pseudo time-series via single-cell transcriptome data. Nat Commun 2017;8:22.

[68] Kowalczyk MS, Tirosh I, Heckl D, Rao TN, Dixit A, Haas BJ, et al. Single-cell RNA-seq reveals changes in cell cycle and differentiation programs upon aging of hematopoietic stem cells. Genome Res 2015;25:1860–72.

[69] Wolpert DH, Macready WG. No free lunch theorems for optimization. IEEE Trans Evol Comput 1997;1:67–82.

[70] Kleshchevnikov V, Shmatko A, Dann E, Aivazidis A, King HW, Li T, et al. cell2location maps fine-grained cell types in spatial transcriptomics. Nat Biotechnol 2022;40:661–71.

[71] Li B, Zhang W, Guo C, Xu H, Li L, Fang M, et al. Benchmarking spatial and single-cell transcriptomics integration methods for transcript distribution prediction and cell type deconvolution. Nat Methods 2022;19:662–70.

[72] Wolf FA, Angerer P, Theis FJ. SCANPY: large-scale single-cell gene expression data analysis. Genome Biol 2018;19:15.
