# Supplementary figures and images for: HemaScope: A Tool for Analyzing Single-cell and Spatial Transcriptomics Data of Hematopoietic Cells
Source: Genomics Proteomics Bioinformatics. 2025 Jan 25;23(2):qzaf002. doi: 10.1093/gpbjnl/qzaf002 (PMC12374577; doi:10.1093/gpbjnl/qzaf002)

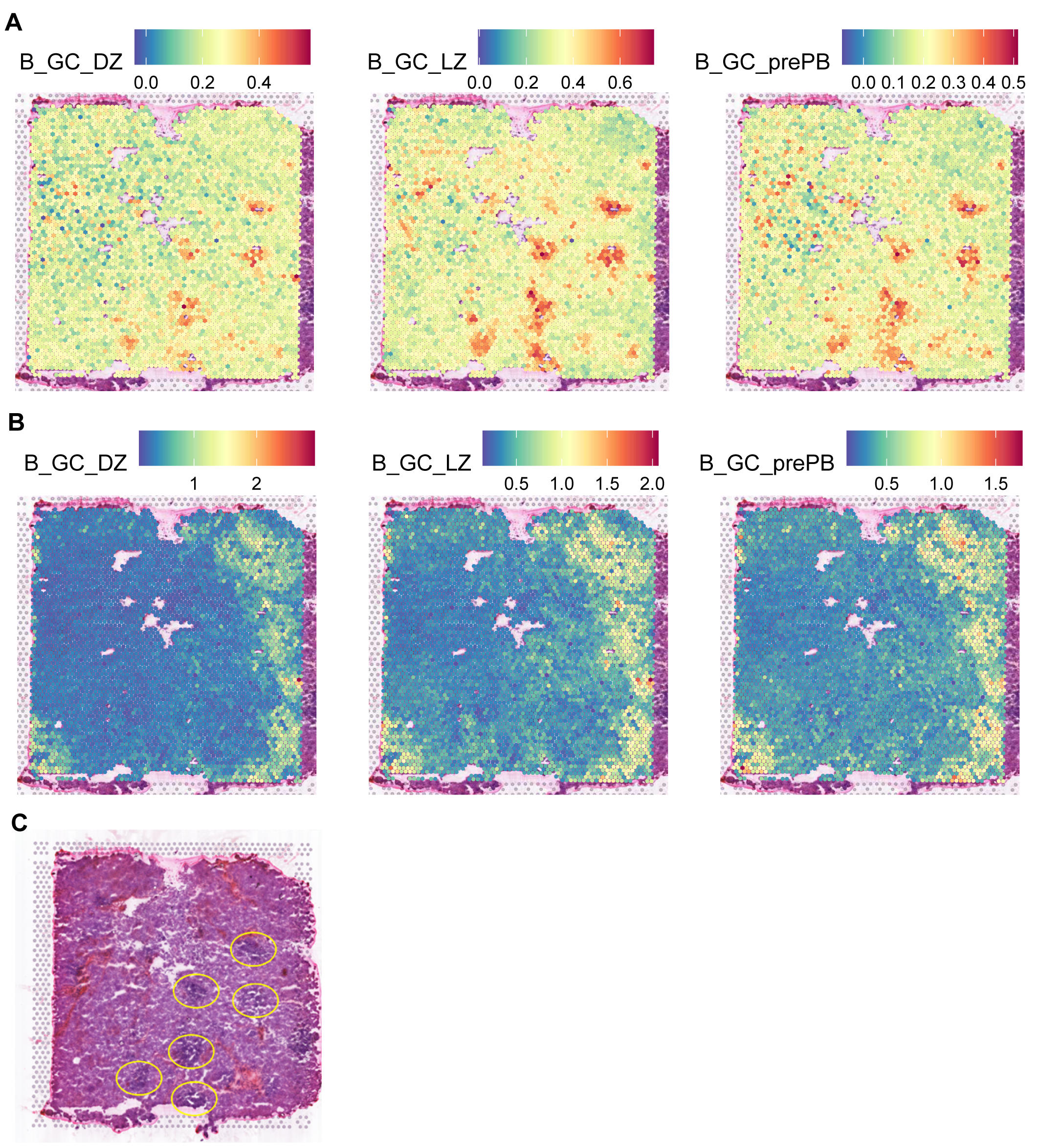

Supplement: qzaf002_Supplementary_Data [file qzaf002_supplementary_data.zip › FigureS7.tif]

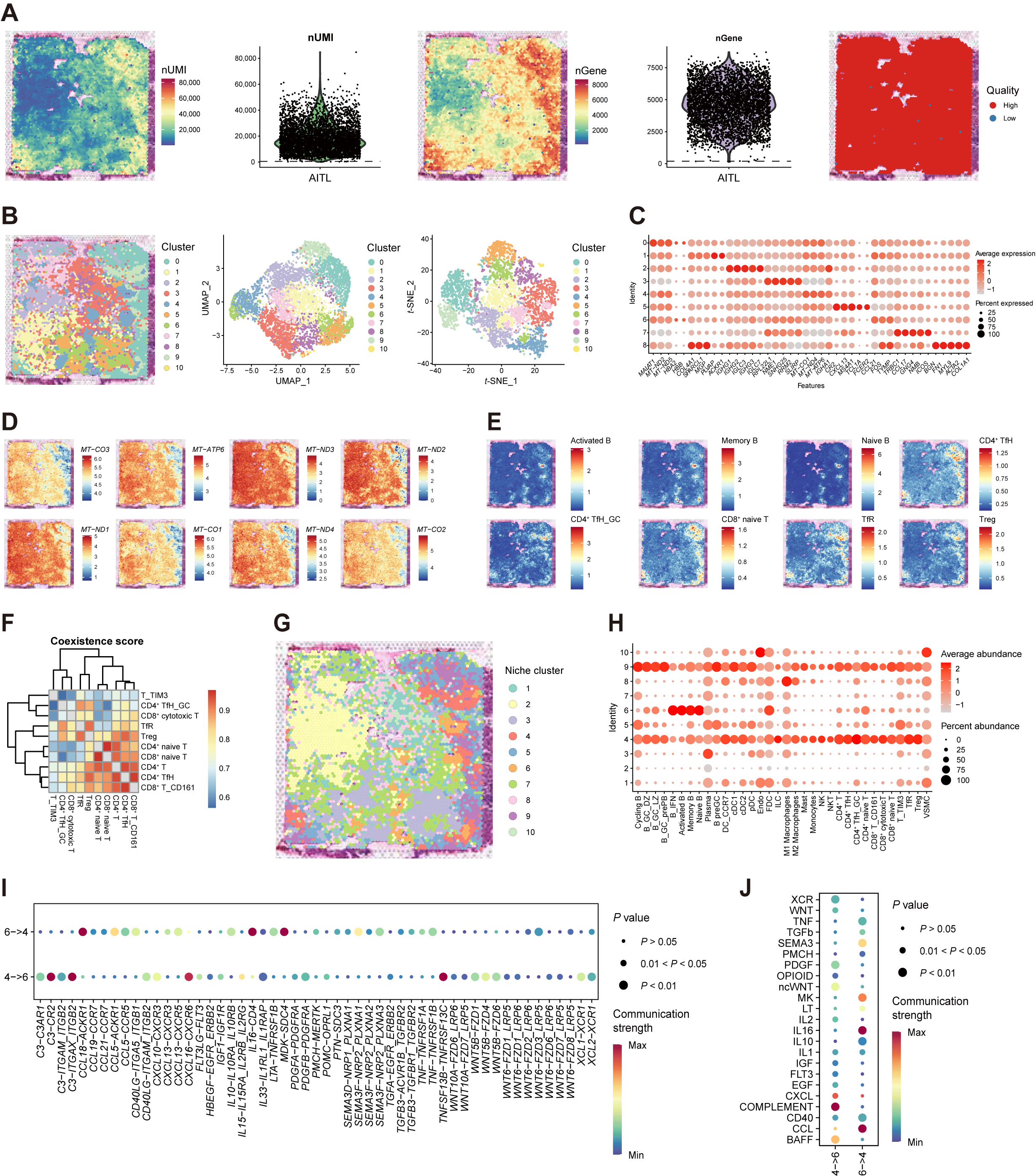

Supplement: qzaf002_Supplementary_Data [file qzaf002_supplementary_data.zip › FigureS5.tif]

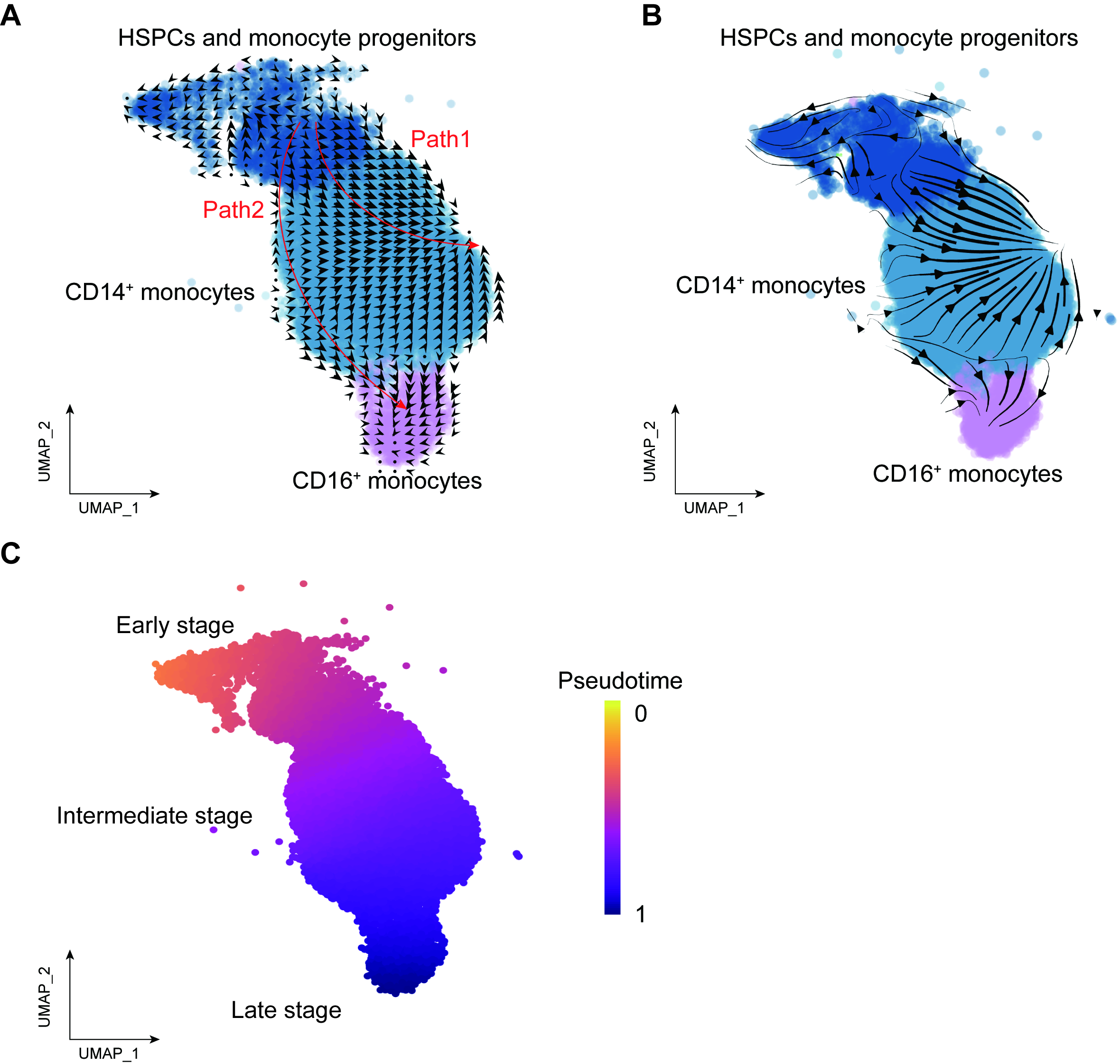

Supplement: qzaf002_Supplementary_Data [file qzaf002_supplementary_data.zip › FigureS6.tif]

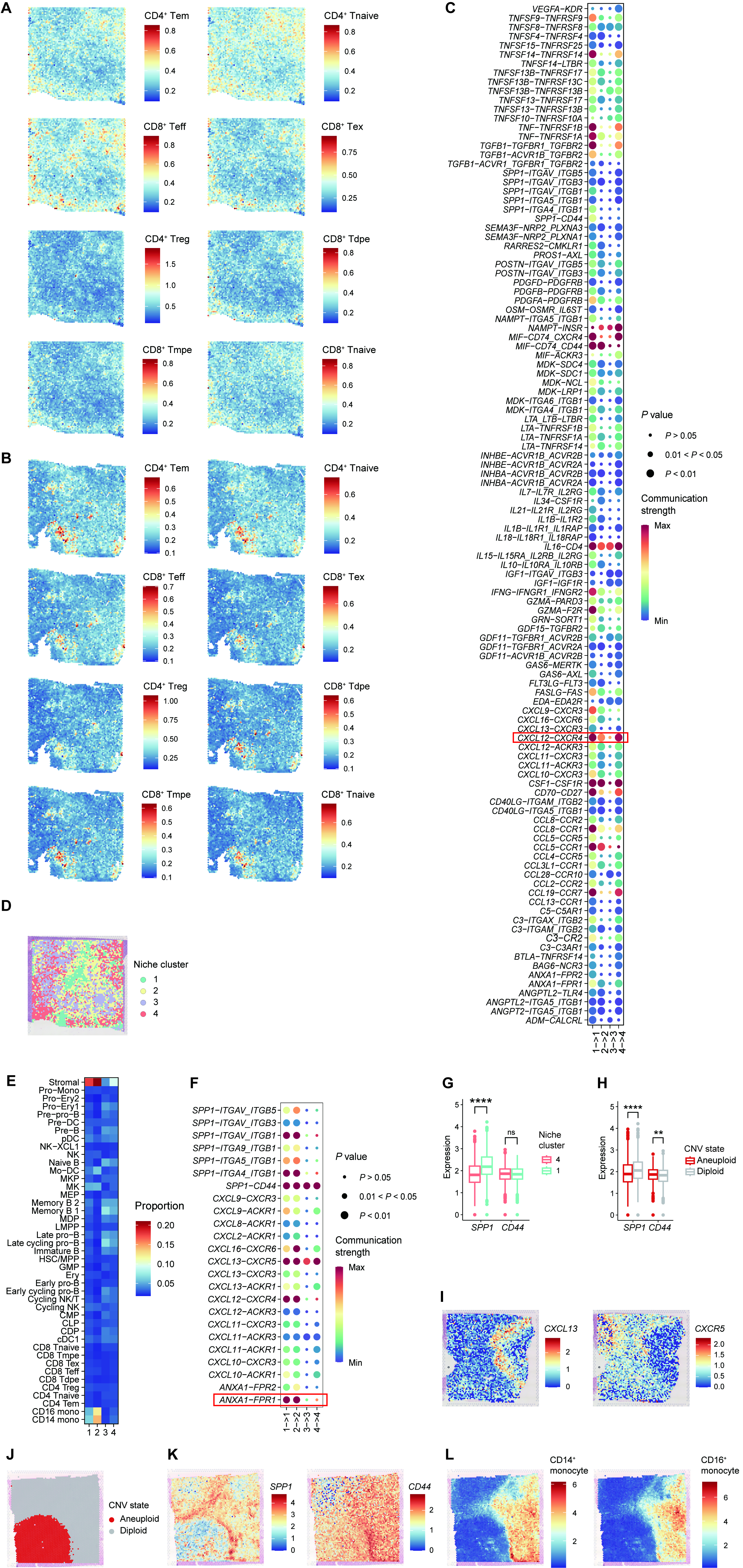

Supplement: qzaf002_Supplementary_Data [file qzaf002_supplementary_data.zip › FigureS4.tif]

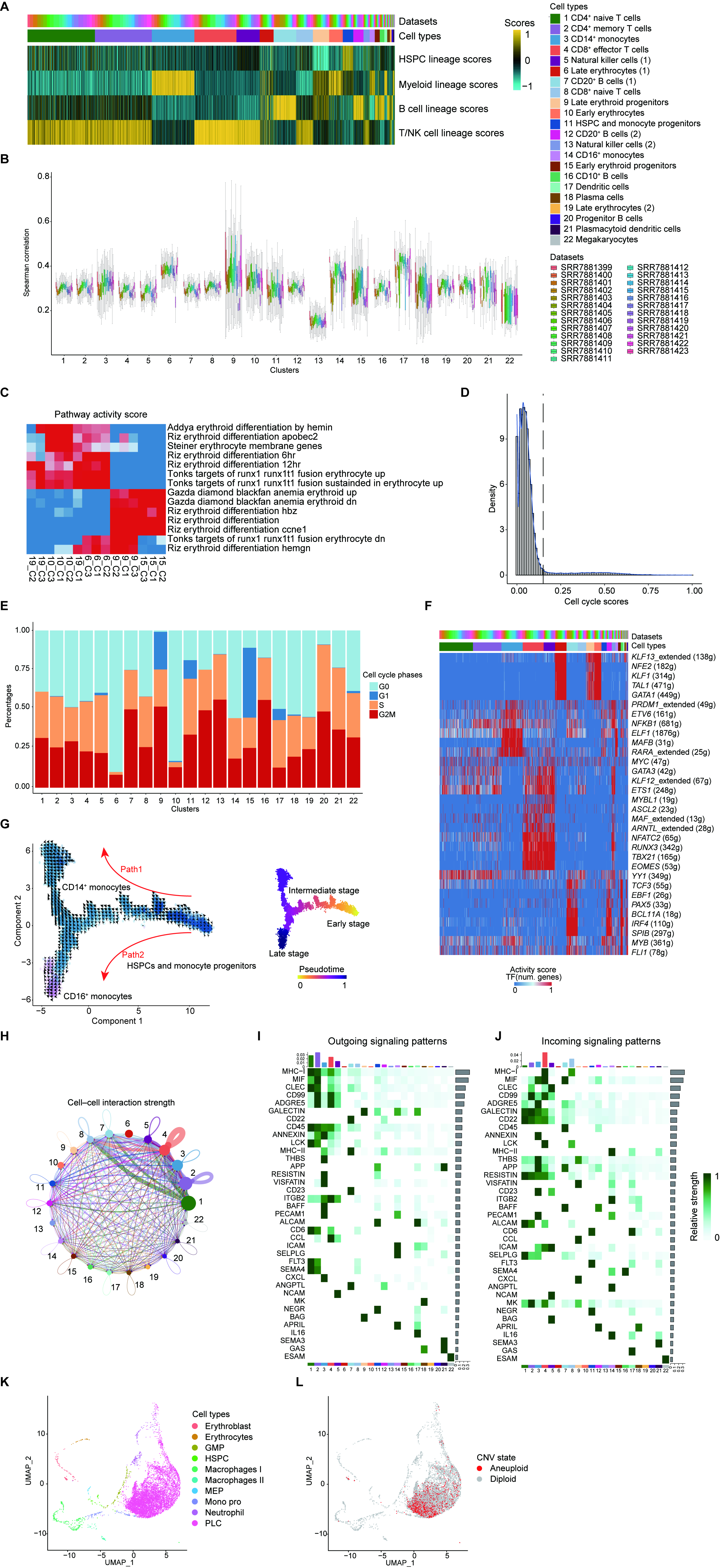

Supplement: qzaf002_Supplementary_Data [file qzaf002_supplementary_data.zip › FigureS3.tif]

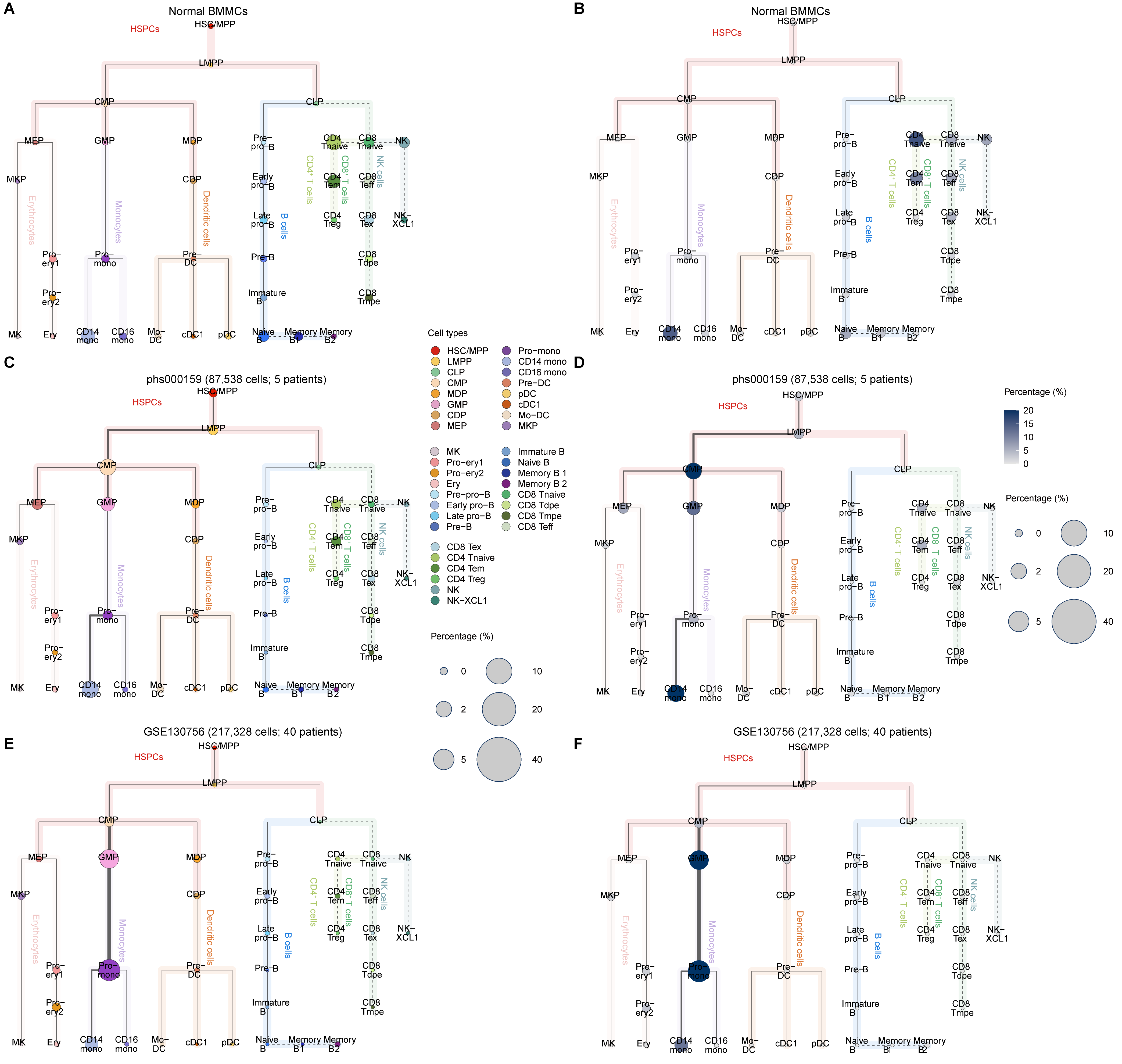

Supplement: qzaf002_Supplementary_Data [file qzaf002_supplementary_data.zip › FigureS1.tif]

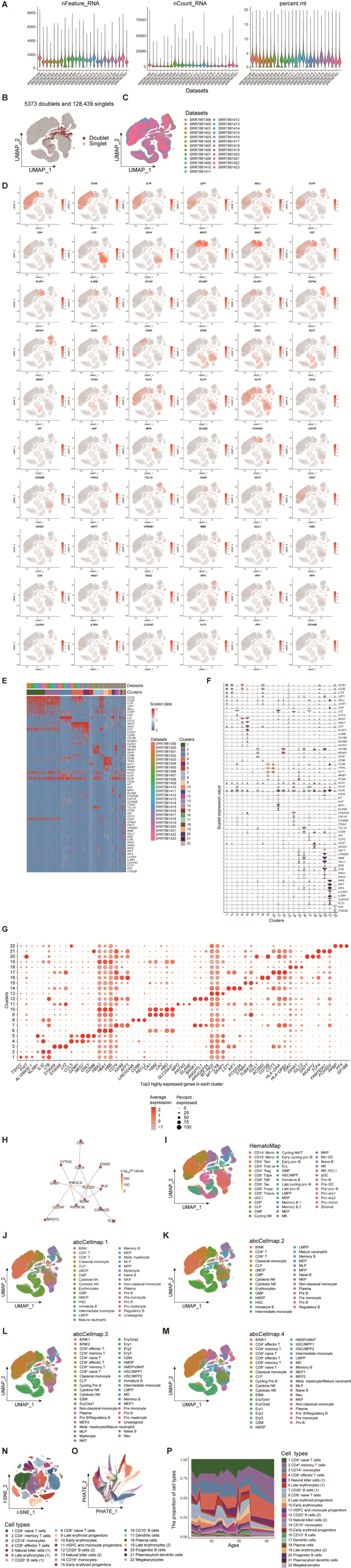

Supplement: qzaf002_Supplementary_Data [file qzaf002_supplementary_data.zip › FigureS2.tif]
